# Singers of the high Arctic; Seasonal acoustic presence of bowhead whales (*Balaena mysticetus*) around Svalbard, Norway

**DOI:** 10.1038/s41598-025-98302-7

**Published:** 2025-04-25

**Authors:** Luca Wams, Kit M. Kovacs, Christian Lydersen, Ulf Lindstrøm, Dag Tollefsen, Heidi Ahonen

**Affiliations:** 1 Norwegian Polar Institute, Fram Centre, Tromsø, 9296 Norway; 2 Institute of Marine Research in Norway, Fram Center, Tromsø, 9296 Norway; 3 UiT The Arctic University of Norway, Hansine Hansens veg 18, Tromsø, 9019 Norway; 4 Norwegian Defence Research Establishment (FFI), Box 115, Horten, 3191 Norway

**Keywords:** Marine biology, Phenology, Animal behaviour, Environmental sciences

## Abstract

The bowhead whale (*Balaena mysticetus*) is an Arctic endemic cetacean that spends its entire life in Arctic waters, living in tight association with sea ice. In this study, passive acoustic data recorded between 2017 and 2022 at six locations around the Svalbard Archipelago, Norway, were analysed for the seasonal and spatial vocal occurrence of this endangered bowhead whale stock, using a combination of Long-Term Spectral Averages and visual/auditory verification. The results were subsequently correlated to seasonal patterns in environmental conditions. Bowhead whale vocalizations were detected at all study sites, but there was significant variation between locations and years in acoustic presence and singing behaviour. Consistent, high detection rates of singing west and east of Svalbard suggest that these sites serve as key areas for overwintering and breeding. The lower and fluctuating detection rates north of Svalbard suggest that this area serves as a movement corridor between regions. Significant positive correlations between bowhead whale acoustic activity and high sea ice concentrations emphasizes their ice-affiliated nature. This study provides novel information on the distribution and habitat use of bowhead whales in the Svalbard area and raises concern for the future of this population, in an Arctic that is experiencing rapid sea ice habitat loss.

**Supplementary Information:**

The online version contains supplementary material available at 10.1038/s41598-025-98302-7.

## Introduction

The Arctic features some of the most unique and remote ecosystems on the planet and is home to some highly specialized species of marine mammals. This region is currently undergoing extreme environmental change due to climate change^1,2^, which presents a high risk for all Arctic endemic marine mammals, including bowhead whales (*Balaena mysticetus*)^3^. Bowhead whales are the only baleen whales that spend their entire life in Arctic waters, and they possess behavioural, physiological and morphological adaptations to live within the region’s harsh climate^4^. This species is the longest-lived cetacean and exhibits a strongly conservative life-history strategy. Their large body size and thick blubber layer are essential for insulation from the cold and for energy storage^5^, which is important given that food availability is highly seasonal^6^. Furthermore, they lack a dorsal fin and can use their massive head to break through sea ice up to 60 cm thick for breathing when leads or ponds are not accessible. These adaptations allow them to occupy areas with high ice concentrations^7^. Sea ice habitats provide them with a spatially extensive environment that is virtually disease-free and it is low in competition, while offering a seasonally rich food supply^8^, as well as shelter from storms and predation by killer whales (*Orcinus orca*)^9^.

Spitsbergen bowhead whales (also known as the East Greenland-Svalbard-Barents Sea stock) are one of four distinct bowhead whale populations. This population inhabits ice-covered waters extending from East Greenland through the Svalbard area and into the western parts of the Russian Arctic^10^. Historically, this was one of the largest bowhead whale populations, estimated to number well over 50,000 adult individuals in 1611^11^. However, after extensive exploitation this population was deemed ‘virtually extinct’ by 1911, with only tens of individuals estimated to be left^12^. In recent decades, several studies have suggested that this population is somewhat larger than previously thought^8,13,14^. The current size of the Spitsbergen population is likely to comprise at least several hundred individuals, although data are still lacking for a meaningful trend assessment^10^. Unlike other bowhead whale populations that have traditional seasonal migration patterns tracking the ice edge northwards in summer and southwards again when ice formation starts in late autumn or early winter^15–17^, the Spitsbergen population lacks a clear migratory pattern. Some individuals spend the winter in almost completely ice-covered areas at the northernmost latitudes of their range, up to hundreds of kilometres inside the ice edge^18^. It is not known why their migratory behaviour differs so radically from their conspecifics in other populations^10^. Possible factors that might play a role in the habitat preference of the Spitsbergen bowhead whales include seasonal food availability^19^, sea ice conditions and their history with extensive human exploitation; animals that showed a strong preference for ice-affiliation were those that survived commercial whaling^10^.

To understand more about bowhead whale habitat selection, behaviour and distribution, several studies have been performed using sighting data^20,21^ and satellite tracking^6,10,18,22^. Satellite tracking documents an individual’s movement over long distances, but this method can be constrained by logistical and financial costs^23^. Visual surveys can be limited through accessibility in ice-covered areas, as well as extreme seasonal variation in light and surface weather conditions^24^. Passive Acoustic Monitoring (PAM) compliments these other methods for monitoring highly mobile marine mammals across long time periods, by providing a non-invasive, low-cost method to detect and record their vocalizations via the deployment of hydrophones. Bowhead whales often reside in inaccessible, ice-covered habitats but they have an extensive and varied acoustic repertoire, making them excellent subjects for PAM^17,25,26^. Stafford et al. (2012) classified bowhead whale vocalizations into three broad types; simple calls, call sequences and songs. Simple calls are described as low frequency moans, with most of their acoustic energy below 500 Hz. They are produced without a distinct pattern and are believed to be used for communication between individuals. These calls occur year-round^27^. Repeated bouts of similar calls are referred to as call sequences^28^, which are most frequently produced in winter^29^. Songs are much more complex than the other vocalization categories; they are broadband acoustic signals with energy reaching up to 5 kHz, consisting of combinations of closely spaced ‘song notes’ which can be repeated for hours at a time^27,30^. Songs are produced seasonally and are thought to be produced by male bowhead whales, as an acoustic display to compete with rival males and attract candidate mates^31^.

**Fig. 1:**
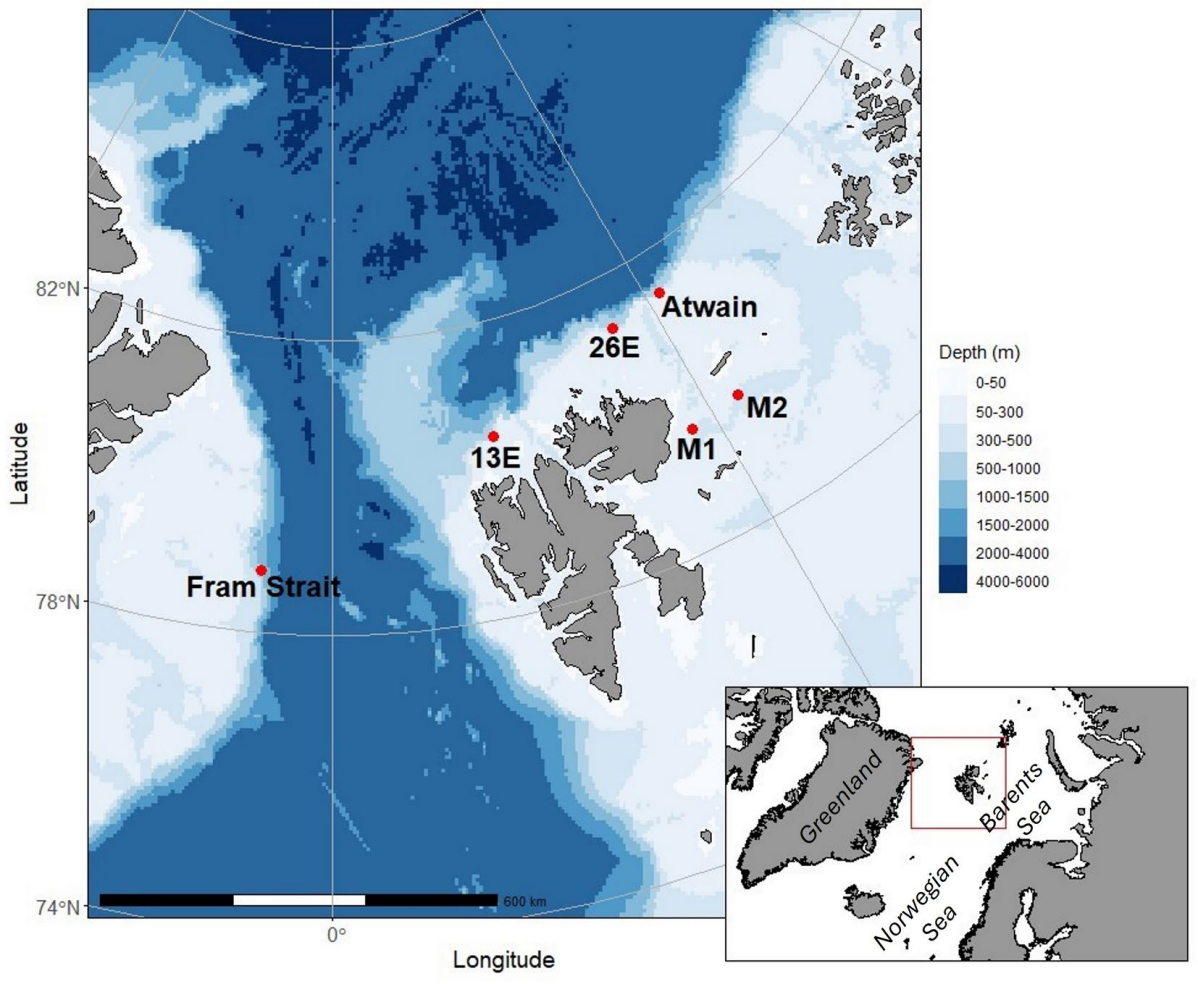
Study area and mooring locations bearing hydrophones around the Svalbard Archipelago, indicated by the red dots. Bathymetric dataset from GEBCO 2023 under-ice bathymetry, accessed via: https://www.gebco.net/data_and_products/gridded_bathymetry_data/.

PAM has become a useful method to reveal more about bowhead whales throughout the Arctic, e.g., in western Greenland^32^, western Fram Strait^25^ and through the Bering Strait^17^. Monitoring populations and investigating correlations between their occurrence and environmental factors is becoming increasingly important in the context of climate change and the rapid environmental change that is taking place in the Arctic. In this study, the acoustic presence of Spitsbergen bowhead whales is investigated across regions west, north and east of Svalbard during autumn, winter and spring. Passive acoustic data from six different locations (Fig. 1) were analysed for bowhead whale acoustic presence and singing behaviour. In addition, the correlation between both acoustic presence and song presence and environmental factors across seasons were investigated to better understand the potential drivers influencing the distribution of Spitsbergen bowhead whales.

## Results

### Acoustic detection

**Fig. 2:**
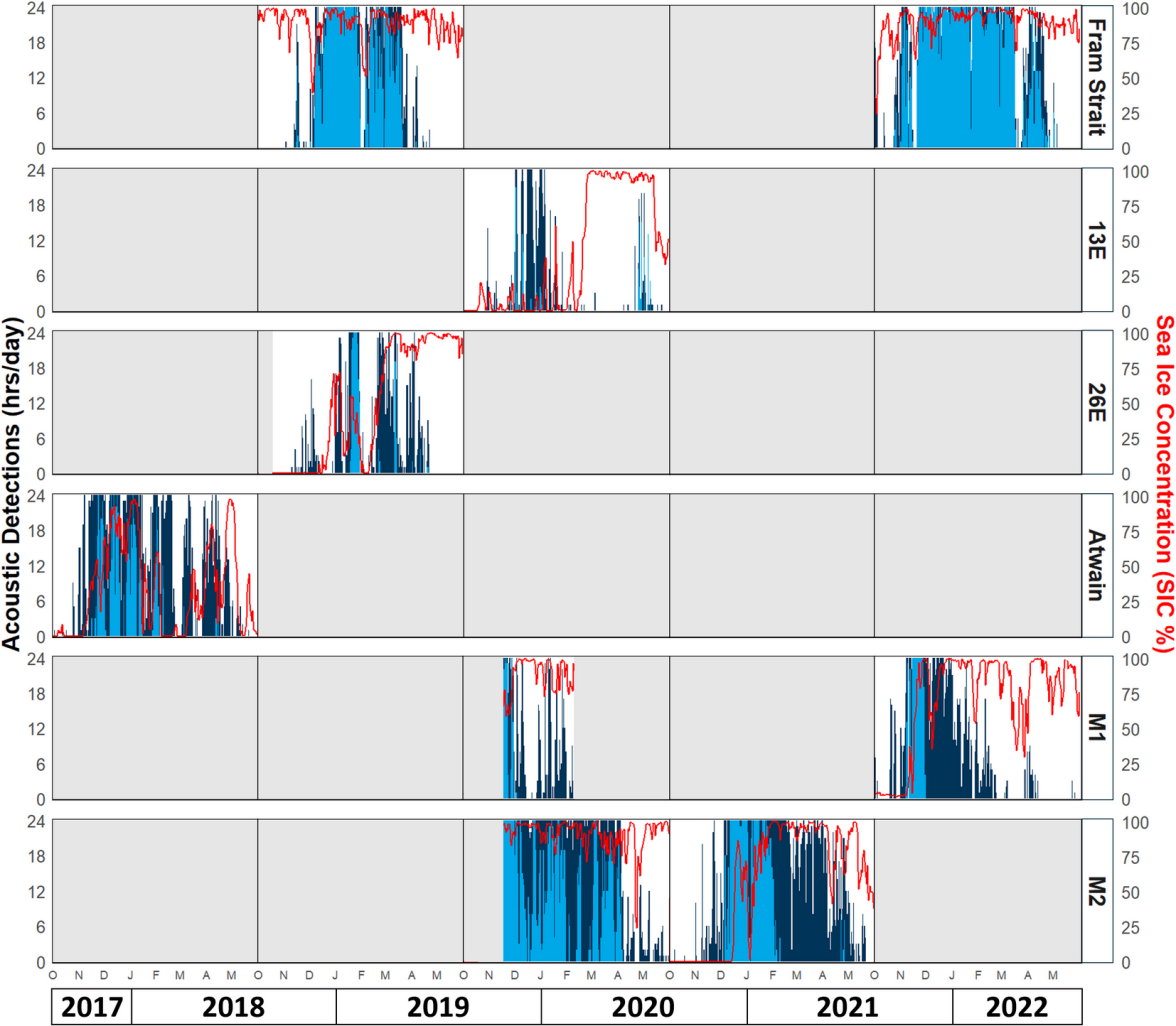
Daily acoustic detections for nine sampling periods across six study locations, in relation to sea ice concentration (%) (red line). Daily counts of acoustic presence (dark blue) and song presence (light blue) for bowhead whales are stacked (song detections also count for bowhead whale acoustic presence). Only the analysed period, from October to May, is shown on the x-axis. More detailed graphs on acoustic detections per study period can be found in Appendix A.

**Fig. 3:**
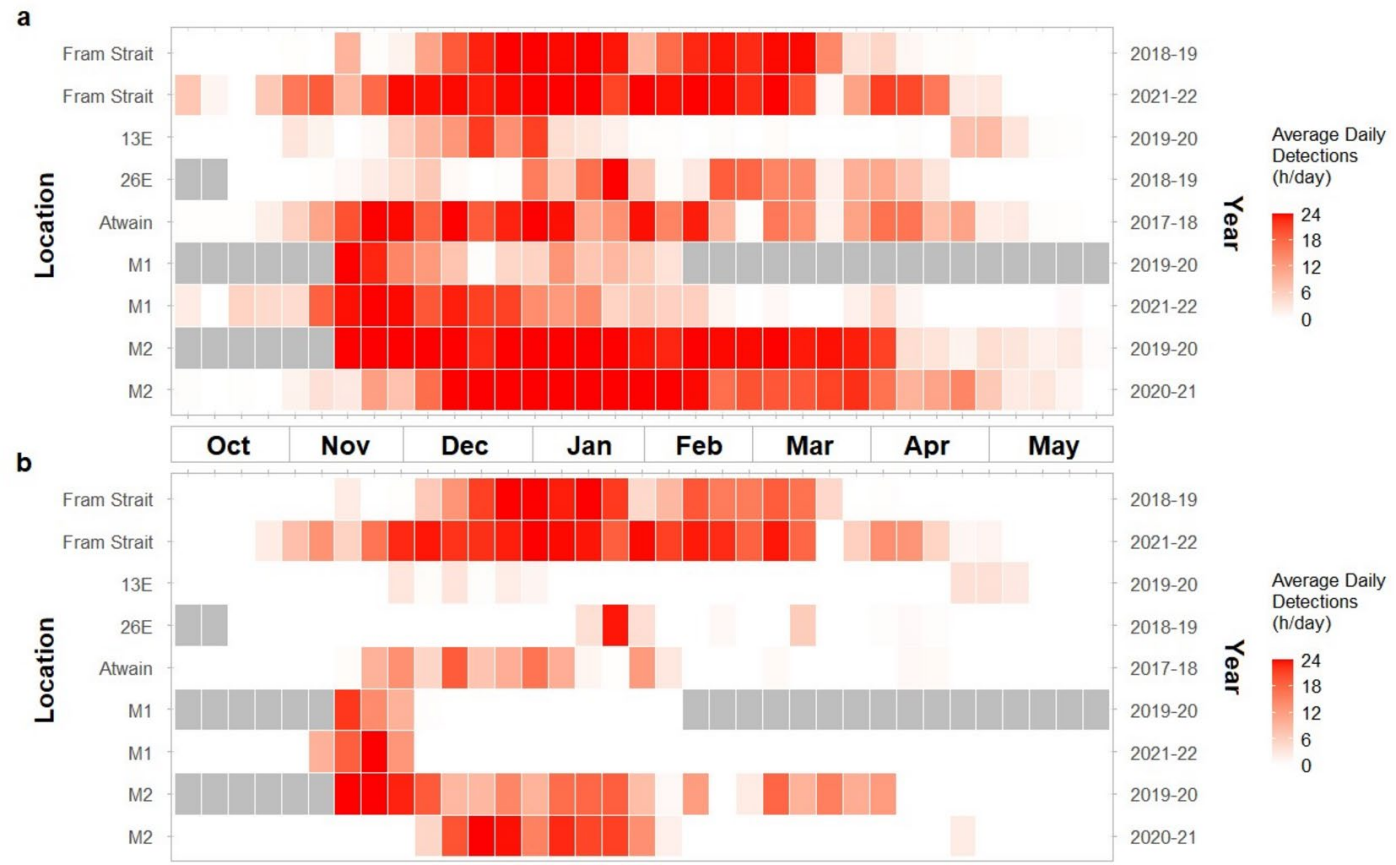
Average daily detections of (**a**) bowhead whale acoustic presence and (**b**) bowhead whale song presence for each sampling period. Colour intensity indicates the average daily detections (0–24) per week. Grey boxes indicate weeks without available acoustic data.

Bowhead whale acoustic presence and song presence were detected in all nine sampling periods, across each of the six study locations (Fig. 2). In total, 20,617 h containing bowhead whale acoustic presence (either song or other vocalizations) were detected across all sampling periods (43.8% of recordings), with the highest detection rates at the M2 and Fram Strait moorings (74% and 63% of recordings respectively). Acoustic presence occurred predominantly between November and April (Fig. 3), peaking in December and January. Few vocalizations occurred in October and May. For both sampling periods at Fram Strait, as well as the 2021–2022 sampling period at M1 and the 2020–2021 sampling period at M2, a clear pattern was seen with vocal activity increasing until a period of relatively constant high presence was reached, followed by a gradual decrease. At 13E, 26E and Atwain, vocal activity was more sporadic, with many gaps in detection.

Bowhead whale song was detected in 9,776 h across all sampling periods (20.8% of recordings). While the predominant singing period varied across locations and years, most of the hours containing song were registered in December and January; little to no song was detected in October and May (Figs. 2 and 3b). The earliest song detection occurred on the 25th of October, at Fram Strait in 2021. The latest song presence was detected on the 22nd of May, at 13E in 2020. Both sampling periods in Fram Strait showed a relatively constant period with high singing activity mid-winter. Some interannual variation can be seen in the duration and intensity of singing; for example, detection rates in 2021–2022 were higher than those in 2018–2019 (Fig. 2). The locations 13E, 26E and Atwain, which are all located north of Svalbard, showed variable patterns in song presence. Compared to Fram Strait, much less singing was detected at these locations, with little or no consistent singing period (Fig. 2). Furthermore, large intervals between days with song were found; for example, at 13E there is a period of 115 days without singing between two detections of song. For both locations in the north-eastern part of Svalbard, M1 and M2, data was analysed for two sampling periods. At M1, all song detections occurred within the same four-week period in November – early December, in both sampling periods. The two sampling periods at M2 showed varying patterns of song presence; in 2019–2020 song detections were spread from mid-November until early April, whereas in 2020–2021, a more concentrated singing period was observed from December to early February (Figs. 2 and 3b).

### Presence and song in relation to environmental cues

**Fig. 4:**
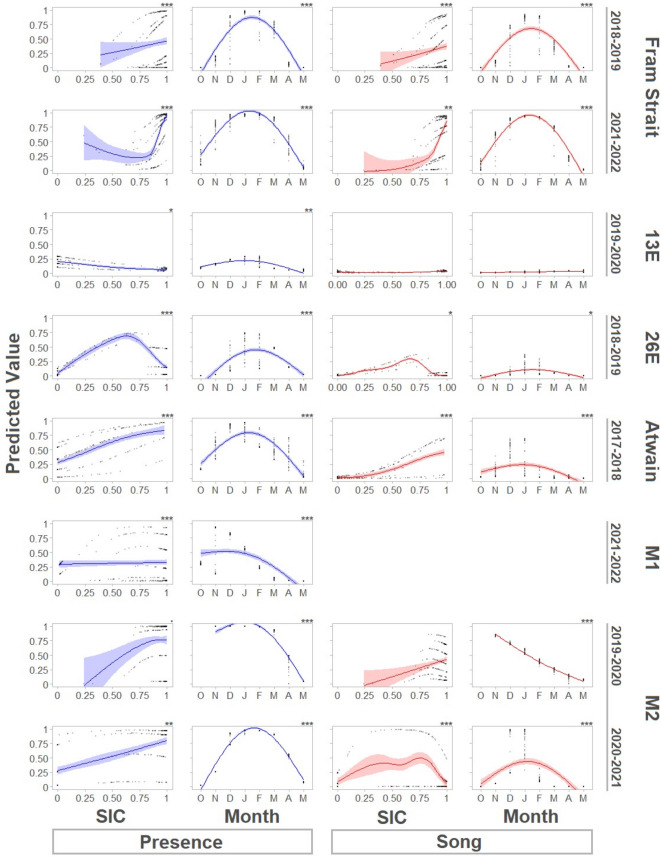
Effects of Sea Ice Concentration (SIC) and month on predicted acoustic presence and predicted song presence of bowhead whales for each sampling period. Predicted values were derived from Generalized Additive Models (GAMs) and plotted as dots. Blue lines represent estimated splines for predicted acoustic presence, red lines represent estimated splines for predicted song presence. These splines illustrate the smoothed relationship between the predictors (SIC and month) and the predicted values. Shaded areas represent the 95% confidence intervals.

During both the 2018–2019 and 2021–2022 sampling periods at Fram Strait, sea ice concentrations (SIC) were consistently high (Fig. 2, Appendix Fig. A1). The mooring remained ice-covered and sea surface temperatures (SST) ranged between − 1 and − 1.9 °C. During the period from November until March, some gaps in vocal presence can be seen in both sampling periods, some of which were correlated with periods of lower ice concentrations. Zooplankton densities and chlorophyll-a biomasses were very low throughout both sampling periods. Significant effects of SIC and month on both acoustic presence (R^2^ = 0.73) and song presence (R^2^ = 0.69) were found in both sampling periods (Fig. 4). Significant positive correlations between SIC and acoustic presence and song presence were detected for ice concentrations of approximately 80% and higher. The effect of month fit a bell curve, with peaks for acoustic presence and song presence in January.

At 13E, SIC was relatively low from October until mid-February, with the mooring mostly being surrounded by open water, near the ice edge (Fig. 2, Appendix Fig. A2). Bowhead whale vocalizations occurred sporadically and showed little correlation to the environmental variables at this site. From mid-February, SST dropped and SIC increased to almost full ice cover, only to decrease again mid-May. During this time, a small cluster of vocalizations, mostly songs, occurred at the end of April, again with little correlation to the environmental variables. Small but significant effects of SIC and month on acoustic presence were found (R^2^ = 0.09; Fig. 4). However, no significant effects were found for song presence. A slight negative correlation was found between SIC and acoustic presence at 13E.

At 26E, ice formation did not start until mid-December, when some sporadic acoustic presence was detected (Fig. 2, Appendix Fig. A3). In late December and January, SST dropped and two peaks in ice concentration coincided with increased acoustic presence and a period of song presence. In early February, SST rose and SIC decreased, corresponding with reduced acoustic activity. From mid-February, SIC increased again and the mooring became situated deep within the ice for the remainder of the sampling period. During this time, sporadic vocalizations occurred until mid-April. Throughout the whole sampling period, zooplankton densities and chlorophyll-a biomasses remained low. Effects of SIC and month on both acoustic presence (R^2^ = 0.49) and song presence (R^2^ = 0.16) were significant (Fig. 4). A positive correlation was found between SIC up to approximately 60% ice cover, and both acoustic presence and song presence. The effect of month on acoustic presence and song presence was bell-shaped, peaking in January.

During the entire sampling period at Atwain, SIC fluctuated markedly (Fig. 2, Appendix Fig. A4). Acoustic presence and song presence also fluctuated, with acoustic detections during both periods of high and low SIC. Zooplankton densities and chlorophyll-a biomasses were close to zero for the entire sampling period. Effects of SIC and month on acoustic presence (R^2^ = 0.47) and song presence (R^2^ = 0.51) were significant (Fig. 4). Acoustic presence and song presence were positively correlated with SIC, and a seasonal peak in both acoustic presence and song presence occurred in January.

During the 2019–2020 sampling period at M1, SIC increased in late-October and remained high throughout most of the remaining period, resulting in continuous ice cover over the mooring (Fig. 2, Appendix Fig. A5). In 2021–2022, SIC started to increase slightly later, in mid-November. The singing period coincided with increasing SIC. Whether this was the case for the 2019–2020 sampling period cannot be determined because no acoustic data were collected before mid-November, at which time the ice was already established over the mooring. Significant effects of SIC and month on acoustic presence were found for the 2021–2022 sampling period (R^2^ = 0.69) (Fig. 4), with a small positive effect of SIC on acoustic presence and peak acoustic presence in November and December, followed by a gradual decline until mid-April.

In the 2019–2020 sampling period at M2, SIC increased in mid-October and remained consistently high throughout the rest of the sampling period (Fig. 2, Appendix Fig. A6). In 2020–2021, SST remained high for much longer, and SIC increased later in the season (mid-December). This corresponded with a later start of the singing period in 2020–2021 compared to 2019–2020. Significant effects of SIC and month on acoustic presence (R^2^ = 0.83) and song presence (R^2^ = 0.65) were found (Fig. 4). SIC had a positive effect on acoustic presence for the 2019–2020 sampling period, however for the 2020–2021 sampling period no significant relationship was established. A significant but fluctuating effect of SIC on song presence was found for 2020–2021, but this was not the case for 2019–2020. In the 2020–2021 sampling period acoustic presence displayed a bell curve with peak acoustic presence and song presence in January. In 2019–2020, acoustic presence peaked in January, while song presence had a linear negative relationship with month from November to May.

## Discussion

This study provides new insights into the seasonal and spatial trends of acoustic presence and singing behaviour of Spitsbergen bowhead whales. Acoustic presence and songs were detected during each of the nine sampling periods, with notable differences between the years and study locations in terms of the duration and intensity of their acoustic presence, as well as the patterns seen in the presence of songs. The most constant and intense vocal activity was found in Fram Strait and at the north-eastern parts of the Svalbard Archipelago, especially at M2. According to Stafford et al. (2012) such persistent acoustic presence indicates that numerous bowhead whales were present near these sampling sites throughout the winter^27^. In Fram Strait, these findings are in accordance with documented acoustic detections of bowhead whales between 2008 and 2014^25^. Since bowhead whale songs are produced by males in connection with breeding, our findings further strengthen the theory that the western part of Fram Strait is a key breeding area for the species. Somewhat fewer vocalizations were detected during autumn and spring in this study, compared to the previously documented acoustic activity at this location, which might be due to the limited ability to detect faint or short simple calls in the LTSA’s. At M2, the high levels of acoustic presence and song presence were similar to previous manual detections of bowhead whale vocalizations in this area^33^, which utilised the same acoustic data as the 2019–2020 sampling period presented in this study. The high degree of similarity between results from manual and LTSA detections, supports that the latter, more time-efficient method for detecting bowhead whale acoustic presence is functional. Furthermore, the high acoustic presence during both sampling periods suggests that this site is another important area for bowhead whales. In addition, based on the high detection rate of songs at this site, it likely represents the discovery of a second breeding area for the Spitsbergen population. The findings in Fram Strait and at M2 stand in stark contrast with the acoustic detections in the area north of Svalbard, where a much more sparse and variable acoustic presence was found. Especially at the locations 13E and 26E, sites not previously studied with respect to bowhead whale acoustic presence, detections occurred only sporadically, with little indication that bowhead whales were present in these areas for any extended periods of time. Detection rates at Atwain were higher, although the patterns in acoustic presence and song remained irregular, with periods of high vocal activity interrupted by brief periods without vocalizations.

The six study locations encompassed a wide range of environmental conditions and covered a large part of the geographical range of Spitsbergen bowhead whales. The Fram Strait mooring was situated on the continental shelf slope between Greenland and Svalbard, where cold and relatively fresh, ice-filled water is exported out of the Arctic Ocean via the East Greenland Current, while relatively warm, salty Atlantic water is transported northwards via branches of the West Spitsbergen Current (WSC). This makes Fram Strait a very dynamic region, with heavy sea ice cover being the norm during winter, especially in western Fram Strait where this mooring was located^34^. These environmental conditions, combined with the low amount of anthropogenic disturbance during the winter^25^ make for a highly suitable breeding ground for bowhead whales. This is reflected in the high detection rates and positive correlations between sea ice concentration and acoustic presence at this location. The three study locations north of Svalbard (13E, 26E and Atwain) were situated in a region with a large inflow of Atlantic Water transported northwards via the WSC. Ice conditions at these sites mainly consist of drift-ice and are highly variable due to fluctuating temperatures, as well as the effects of currents and wind on the ice pack^35,36^. These environmental conditions could explain the sparse and sporadic patterns in bowhead whale detections, both in terms of acoustic presence and song detections. The higher detection rate at Atwain compared to 13E and 26E might be partly attributable to the Atwain mooring being deployed slightly further east, in significantly shallower waters, however other environmental parameters could also have played roles. The positive correlations between sea ice and presence at 26E and Atwain suggest that the bowhead whales north of Svalbard still make use of sea ice habitats, despite the fluctuating and unpredictable ice conditions in these areas. The M1 and M2 moorings were situated in the northern part of the Barents Sea, and are affected by both Atlantic Water from the WSC, as well as cold, ice-filled waters drifting southwards from the Arctic Ocean^35^. This results in relatively stable ice cover at these study locations, but with large interannual variability in the timing of when the drift ice arrives and disappears. This is reflected by the interannual variation in acoustic detections at these locations, especially at M2. Here a clear delay can be seen in the onset of song, corresponding to a significant delay in ice cover in 2020–2021, compared to 2019–2020. A more modest acoustic presence was found at M1, compared to M2, perhaps because the M2 mooring was located slightly further off-shore and closer to the coastal polynyas around Franz Josef Land, which are another important area for Spitsbergen bowhead whales based on reported observations from this Russian archipelago^37,38^.

The differences in acoustic presence and singing patterns observed across the six study locations, combined with the environmental differences between these regions, suggest that the area north of Svalbard serves as a movement corridor between the regions west and east of Svalbard, which seem to be preferred areas for Spitsbergen bowhead whales. Tracking studies have shown that individuals in this population spend their winters in the northern-most latitudes of their geographical range, with some tagged individuals having moved from Fram Strait over to Franz Josef Land in Russia^10^. Recent tracking data shows more individuals moving in east-west directions through the area north of Svalbard, further supporting this movement corridor theory (Nowak, Lydersen, Trites, Heide-Jørgensen, Kovacs, unpublished data).

Both acoustic presence and song presence in this study peaked in mid-winter at most of the study locations, with a gradual increase until January, followed by a subsequent gradual decrease toward the spring. At M2 in the 2019–2020 sampling period, the effect of month on song presence was not bell-shaped but song presence declined gradually over the course of the sampling period. This could be attributed to the fact that this sampling period did not span the full eight months between October and May; it lacked the initial autumn period, when acoustic presence generally was expected to commence. It is important to note that although simple bowhead whale calls can occur year-round, this study focused on the fall, winter, and spring months when bowhead whales are most acoustically active and when song is common. Therefore, references to seasonal presence in this study specifically refer to patterns observed between October and May and do not account for potential presence outside of this period.

Significant correlations were found between sea ice concentration and both the presence of bowhead whales and how much singing they displayed. These findings fit well with the ice-affiliated nature of bowhead whales; periods of song coincided with periods of sea ice formation or high sea ice cover. However, when interpreting acoustic presence or absence as a response to sea ice conditions, it is important to consider that bowhead whales might have been present but vocally inactive. For example, Stafford et al. (2012) suggested bowhead whales may produce fewer vocal signals in areas of low sea ice concentration, due to the potential risk of killer whale predation^27^.

Variation in the strength of the relationship between sea ice concentration and acoustic presence or song presence indicates that there might also be other factors that play a role in determining bowhead whale behaviour and habitat selection. Such factors can include endogenous rhythms^39^, and likely also other environmental cues. While sea surface temperature and distance to the ice edge were excluded from the statistical analyses due to high collinearity with sea ice concentration, they likely do contribute to bowhead whale acoustic phenology. The distance to the ice edge can be difficult to interpret, since sea ice extent can change rapidly in drift-ice areas, and it also differs greatly regionally. Spitsbergen bowhead whales likely also make use of coastal polynyas and flaw lead systems^10^, which are not concretely identified in sea ice extent data. Other environmental variables such as oceanic currents, wind, bathymetry, predation risk, or anthropogenic disturbances such as vessel presence or seismic blasting might also play a role in where bowhead whales are found or how vocally active they are at a given time. The satellite-derived chlorophyll-a biomass and zooplankton densities were low and too consistent to contribute to explaining the variance in acoustic presence and song presence of bowhead whales in this study. However, it is important to note that remote sensing satellite data is limited by sea ice and cloud cover, which potentially affected the accuracy of these estimates^40^. While it is assumed that bowhead whales feed primarily during the months with daylight, some studies have provided evidence of winter feeding in other bowhead whale populations^41–44^. Since some copepod species occupy intermediate depths during winter in the Barents Sea^45^, this could facilitate some winter feeding for Spitsbergen bowhead whales as well. Furthermore, the advection of Atlantic zooplankton has a large peak of advected biomass (mainly consisting of copepod species) in December in the northern Barents Sea region^36^, further facilitating potential winter feeding. Therefore, although this study is focused around the winter season and feeding is not considered to be a primary activity during this time, prey availability might still play a role in bowhead whale habitat preference.

## Conclusion

This study has provided new insight regarding the seasonal acoustic presence of Spitsbergen bowhead whales around the Svalbard Archipelago. Bowheads were detected at all recording sites, with the highest detection rates at locations west and east of Svalbard. The predominance of song during the winter months at specific sites highlights the importance of these key areas for bowhead whale overwintering and likely breeding. Furthermore, this study demonstrated significant correlations between sea ice concentration and bowhead whale presence and singing behaviour. Future work should be continued with PAM in this region, which in combination with satellite tracking could improve our understanding of spatial patterns, potential changes in habitat use and the impacts of environmental variables on bowhead whale distribution. Such knowledge is essential for effective climate change and anthropogenic disturbance mitigation strategies, to ensure the conservation of the Spitsbergen bowhead whale population.

## Methods

### Acoustic data collection

This study used acoustic data collected at six locations around the Svalbard Archipelago (Fig. 1). At four locations (Fram Strait, Atwain, M1 and M2), Autonomous Underwater Recorders for Acoustic Listening (AURAL M2/M3, Multi-Électronique Inc.; HTI-96-MIN hydrophone with recording sensitivity of -164 ± 1 dB re 1 V/µPa) were deployed on oceanographic moorings. These four moorings were deployed and maintained by the Norwegian Polar Institute. At the remaining two study locations (13E and 26E), the acoustic data was collected via M5-V30-370 hydrophones (GeoSpectrum Inc.; hydrophone sensitivity of -164.2 ± 1 dB re 1 V/µPa) attached to AMAR-G3-UD recording instruments (Jasco Applied Sciences). These two moorings were deployed and maintained by the Norwegian Defence Research Establishment. From the acoustic data recorded at the six locations, this study utilized data that was collected in the bowhead whale singing period, which typically starts in late October or early November and ends in April or early May. For the Fram Strait, M1 and M2 locations, two sampling periods were available for analyses, while for 13E, 26E and Atwain only one sampling period was available for each, resulting in a total of nine sampling periods. The complete data set including all nine sampling periods spanned from October 2017 until May 2022. For 26E (2018–2019) and M1 and M2 (2019–2020), the sampling periods did not cover the full 8-month period of interest, but rather started later or ended earlier. Due to the limiting effects of battery life and hard drive capacity, the duty cycles and sampling rates varied slightly between sampling periods. The duty cycles ranged between 6 and 20 min recorded every hour, which is sufficient to assess hourly presence in the singing period, when vocalizations are frequent and often repeated for extended periods^46^. Sampling rates varied between 16 and 128 kHz, which is sufficient to capture the entire bandwidth of bowhead whale vocalizations (20–5000 Hz)^27^. For locations 13E and 26E, the hourly duty cycle of 20 min alternated between two consecutive hours where the recordings were made with a lower sample rate, followed by one hour where the recordings were made with a higher sample rate. Full deployment details for each sampling period are presented in Table 1.

**Table 1:** Mooring metadata.

Location	Recording Start	Recording End	Latitude	Longitude	Deployment Depth (m)	Bottom Depth (m)	Sample Rate (kHz)	Duty Cycle (min/h)	Data Source
Fram Strait	01/10/2018	31/05/2019	78,84 N	5,00 W	81	1022	32	12	1
Fram Strait	01/10/2021	31/05/2022	78,84 N	5,00 W	81	1022	128	6	1
13E	01/10/2019	31/05/2020	80,45 N	13,20 E	840	1050	16 and 32*	20	2
26E	19/10/2018	31/05/2019	81,30 N	26,00 E	1155	1365	16 and 64*	20	2
Atwain	01/10/2017	31/05/2018	81,41 N	31,24 E	55	206	32	12	1
M1	18/11/2019	08/02/2020	79,58 N	28,07 E	60	252	32	12	1
M1	01/10/2021	31/05/2022	79,58 N	28,07 E	60	252	32	12	1
M2	18/11/2019	31/05/2020	79,68 N	32,31 E	67	350	32	12	1
M2	10/01/2020	31/05/2021	79,68 N	32,31 E	67	350	32	12	1

Data sources: 1 = Norwegian Polar Institute, 2 = Norwegian Defence Research Establishment. Deployment depth refers to the depth at which the hydrophone was positioned on the mooring line. Duty cycle refers to the duration of each recording in minutes, recorded from the start of every hour. * indicates alternating sampling, where two hours with a lower sample rate were followed by one hour with a higher sample rate.

### Acoustic data analysis

Long-Term Spectral Averages (LTSA’s) spanning over 12 h of acoustic data were used for the detection of bowhead whale vocalizations in each individual audio file. This method has been used previously for investigating the presence of bowhead whale vocalizations^17^. Due to a limited detection ability for faint or short simple calls in LTSA’s, combined with the challenge of distinguishing bowhead whale vocalizations from overlapping humpback whale (*Megaptera novaeangliae*) vocalizations, this study focused its acoustic data analysis solely on the bowhead whale singing period. This period was determined to be between October and May, based on reported bowhead whale singing behavior^27,46^. For study periods where data were available prior to October, summer months were explored and no bowhead whale song was found.

**Fig. 5:**
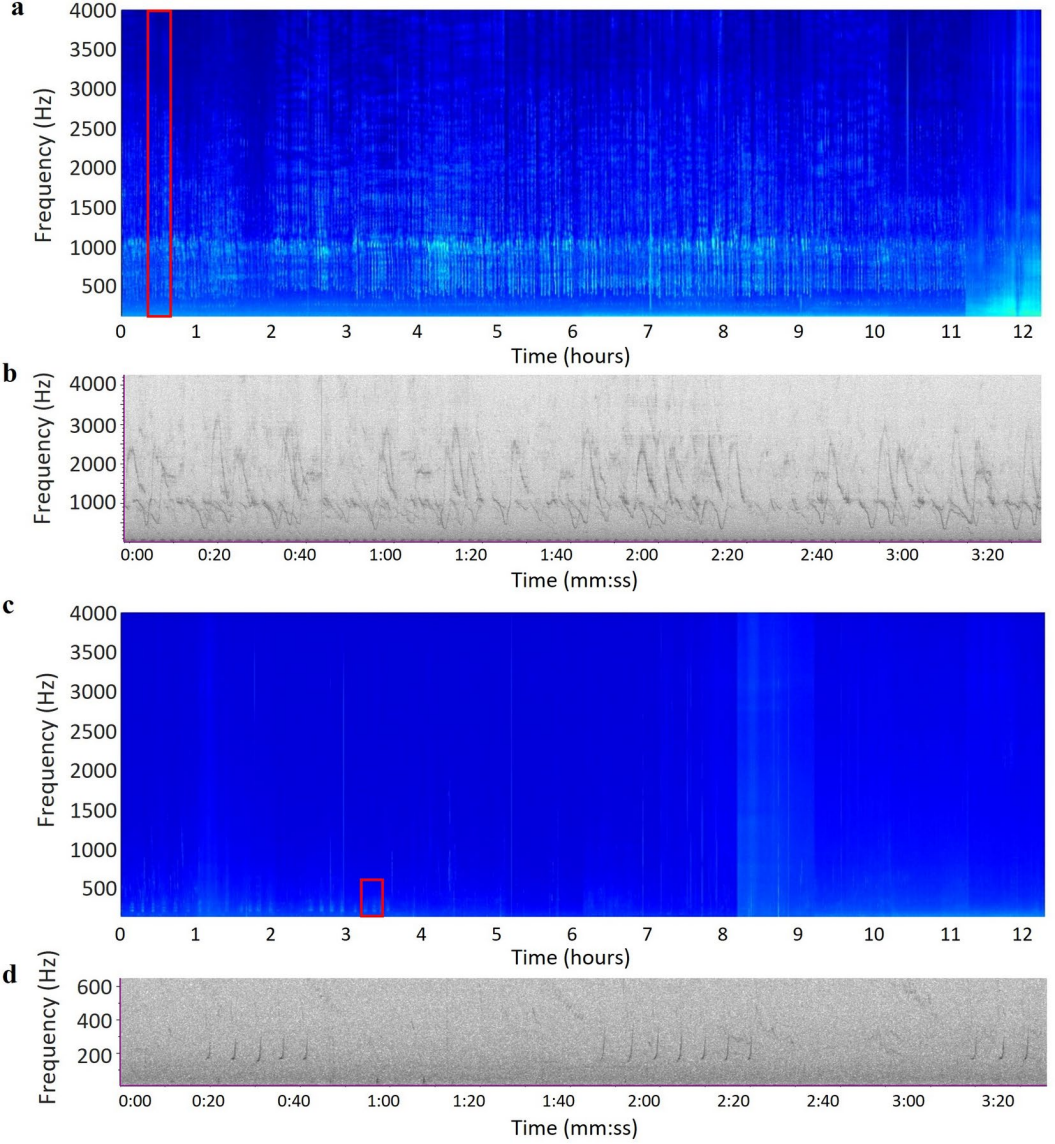
Examples of (**a**) a Long-Term Spectral Average (LTSA) depicting 12 h of bowhead whale song, recorded at Fram Strait, 3 January 2022 with (**b**) a more detailed, zoomed-in depiction of the song, and (**c**) an LTSA depicting bowhead whale call sequences in the first 4 h, recorded at M2, 20 April 2020, with (**d**) more detailed depictions of the call sequences. Red boxes in 5a and 5c indicate where zoomed-in spectrograms (5b and 5d) were taken.

The open-source bioacoustics software PAMGuard (version 2.02.07; available at http://www.pamguard.org)^47^ was used to pre-process all acoustic files for LTSA construction. Spectrograms were created with a 4,096 datapoint Fast Fourier Transform (FFT), Hanning windowed with 75% overlap, and with an averaging interval of 5 s. For sampling periods where the duty cycle alternated between high and low frequency recordings, the high frequency audio files were downsampled to match the sampling rate of the low frequency files, using the decimator module in PAMGuard. Additionally, for the sampling period where the sampling rate was 128 kHz, audio files were downsampled to 32 kHz, to reduce processing time and match the frequency range of most other sampling periods. All down-sampling was performed using a low pass IIR Chebyshev filter, with a cut off frequency of 0.8 times the Nyquist frequency. After pre-processing, 12-hour LTSA’s were plotted using MATLAB (version R2023a) with a frequency range of 0–4000 Hz, and visually assessed for the hourly presence of bowhead whale vocalizations. Acoustic presence and song presence were scored separately, to gain a comprehensive understanding of vocal behaviour throughout the singing period. Hours that contained repeated, complex units with at least one broadband component, were scored positive for both acoustic presence and song presence (Fig. 5a). When bowhead whale vocalizations were found that consisted of simple units without broadband components, hours were scored positive for acoustic presence (Fig. 5b). Hours that contained: (1) sounds of overlapping vocalizing marine mammals (e.g. humpback whales in early autumn or bearded seals (*Erignathus barbatus*) in spring); or (2) interfering noise from e.g. ice formation or hydrophone strumming; or (3) sounds of interest that were either too faint or short to be definitively scored as bowhead whale were marked for later manual verification. Verification took place through both visual and auditory assessments of spectrograms in Raven Pro 1.5 (Bioacoustics Research Program, Cornell Lab of Ornithology).

### Environmental data collection

Daily sea ice concentration data were retrieved from sea ice remote sensing datasets of the University of Bremen, a service component of the GMES Polar View project and the Arctic Regional Ocean Observing System (ArcticROOS). Sea ice concentrations were retrieved by applying the ARTIST Sea Ice (ASI) algorithm to satellite-derived microwave radiometer data, obtained through the Advanced Microwave Scanning Radiometer 2 (AMSR-2) sensor. These data were organized into a polar stereographic grid with a spatial resolution of 3.125 km^2^^48^. Sea ice concentrations, ranging from 0 (open water) to 100 (complete ice cover) were computed daily for each sampling period in the Statistical Software R (v4.4.1), using the *sp* (v2.1.4)^49^ and *raster* (v3.6.26)^50^ packages. Based on estimated detection ranges of bowhead whale vocalizations^51^, ice concentrations were averaged over a 30 km radius around each mooring location.

Sea ice extent data were retrieved from the National Snow and Ice Data Center (NSIDC, Boulder, Colorado), where satellite passive microwave-derived data are used for calculating the Sea Ice Index. This Sea Ice Index data has a spatial resolution of 25 km^2^, wherein a grid cell is considered ‘ice’ at ice concentrations of 15% or higher. Therefore, the ice edge is defined by the 15% concentration contour^52^. Daily GeoTIFF files depicting the sea ice extent for the Northern Hemisphere were retrieved for each sampling period. The daily minimum distance (km) from each mooring site to the ice edge was computed in R using the *rgdal* (v1.6.7)^53^ and *raster* packages. These distances were subsequently transformed so that a positive distance to the ice edge indicates an ice-covered mooring, whereas a negative distance to the ice edge means that the mooring was in open water.

Other environmental data were retrieved from reanalysis datasets of the EU Copernicus Marine Service. Mass concentration of chlorophyll-a (mg/m3) was obtained from the Global Ocean Biogeochemistry Hindcast^54^, with a spatial resolution of 0.25° x 0.25°. Mass content of zooplankton expressed as carbon in sea water (g/m2) was obtained from the Global Ocean Low and Mid Trophic Levels Biomass Content Hindcast^55^, with a spatial resolution of 0.083° x 0.083°. Sea surface temperature (°C) was obtained from the OSTIA global sea surface temperature reprocessed product^56^, with a spatial resolution of 0.05° x 0.05°. For all these variables, daily values were retrieved for each study location and sampling period in R, using *sp* and *raster* packages. Similar to the sea ice concentration data, daily means were calculated over a 30 km radius around each mooring location.

### Data exploration and statistical analyses

Before exploring the statistical relationships between the environmental variables and bowhead whale acoustic presence or song presence, visual comparisons between collected environmental data and acoustic detections were conducted. Because the acoustic analyses focused on data from autumn, winter and spring, the levels of zooplankton and chlorophyll-a mass were very low across all study sites, with almost no variation. These variables were therefore only visually inspected and not included in the statistical analyses. To examine the effects of the remaining environmental variables on acoustic presence and singing behaviour, Generalised Additive Models (GAMs) were fitted, using the *mgcv* (v1.9.1)^57^ package in R. Due to the high variability in environmental conditions across the different study locations and initial visual data exploration indicating significant differences in the responses to environmental variables, the GAMs were fitted separately for each location. Furthermore, separate GAMs were used to examine the degree to which the environmental variables influenced either acoustic presence or song presence at each site in each sampling period.

Before fitting the models, the predictors sea surface temperature (SST), sea ice concentration (SIC) and distance to ice edge (DTI) were checked for collinearity, both through the Pearson Correlation Coefficient, using the *stats* (v4.4.1) package in R, as well as using the *vif* function from the *car* (v3.1.2)^58^ package. For most locations, a strong negative correlation was found between SST and SIC, SST and DTI, as well as a strong positive correlation between DTI and SIC. Because high collinearity can affect both model performance and interpretation, only SIC was kept as a predictor variable.

Daily acoustic presence and daily song presence (0–24 h/day) were transformed to daily proportional acoustic presence and daily proportional song presence by dividing the number of hours with respective acoustic and song presence by 24. SIC values were scaled accordingly. The sampling period 2019–2020 at M1 was excluded from the statistical analyses, due to the considerably shorter period with acoustic data available for this sampling interval. Predictors SIC and month were included as smooth terms with a smoothness degree (k) of 3, which was determined as part of the model fitting process. The models were fitted using restricted maximum likelihood (REML) estimation, to ensure robust and efficient parameter estimation. The corAR1 term from the *nlme* (v3.1.164)^59^ package was included in the model, to account for potential autocorrelation. For the locations with two sampling periods, interaction terms between the covariates and the sampling period were included. This allowed for the effects of SIC and month to vary per sampling period, accounting for other uncaptured environmental differences between the two periods. The full models for these locations were specified as:

$$\begin{gathered} acoustic{\text{ }}presence/song{\text{ }}presence\,={\,_{s1}}\left( {SIC,{\text{ }}by{\text{ }}sampling{\text{ }}period} \right){\text{ }} \hfill \\ {+_{s2}}\left( {month,{\text{ }}by{\text{ }}sampling{\text{ }}period} \right)+{\text{ }}sampling{\text{ }}period \hfill \\ \end{gathered}$$

and the full models for locations with a single sampling period were specified as:

$$acoustic{\text{ }}presence/song{\text{ }}presence\,={\,_{s1}}\left( {SIC} \right){\text{ }}{+_{s2}}\left( {month} \right)$$

where _s1_ and _s2_ are the smooth functions being estimated.

During the model fitting process, models were checked for dispersion issues by calculating the ratio of the residual deviance to the residual degrees of freedom. For all locations the model residuals were under-dispersed (0.15–0.56), indicating less variability in the acoustic data than expected by the model. This was most likely caused by zero/one-inflation issues (U-distribution), because the acoustic presence and song presence were heavily inflated with days without any detections, or days with 24 h of detections. To address this issue, models were compared using the Tweedie family^60^, because this is a flexible distribution suitable for zero-inflated data^61^, and using a quasi-family; quasi-Poisson for count data (0–24) and quasi-binomial for proportional data (0–1), because these families add a dispersion parameter to the model^62^. The comparison showed a slightly better model fit when using the quasi-binomial family, therefore this was chosen for the final models.

After fitting the models, predicted values for presence and song were obtained using the predict function from the *mgcv* package, and plotted using the *ggplot2* (v3.5.1)^63^ package. Model outcome details and residual plots can be found in Appendix A.

## Electronic supplementary material

Below is the link to the electronic supplementary material.

Supplementary Material 1

## Data Availability

Bowhead whale acoustic presence data and environmental data used in analyses and figures are deposited in the Norwegian Polar Data Centre: https://doi.org/10.21334/npolar.2024.2f5c1188.

## References

[1] Rantanen, M. et al. The Arctic has warmed nearly four times faster than the Globe since 1979. *Commun. Earth Environ.* **3**, 168 (2022).

[2] Druckenmiller, M. L. et al. The Arctic. *Bull. Am. Meteorol. Soc.* **102**, S263–S316 (2021).

[3] Kovacs, K. M. et al. State of the Arctic marine biodiversity report update: marine mammals. Technical Report, Conservation of Arctic Flora and Fauna, Akureyri, Iceland (2021).

[4] George, J. C. *Growth, Morphology and Energetics of Bowhead Whales (Balaena Mysticetus)* (University of Alaska Fairbanks, 2009).

[5] Rugh, D. J. & Shelden, K. E. W. Bowhead whale: *Balaena mysticetus*. in *Encyclopedia of marine mammals (second edition)* (ed. Perrin, W. F., Würsig, B., Thewissen, J. G. M.) 131–133Academic Press, London, (2009).

[6] Ferguson, S. H., Dueck, L., Loseto, L. & Luque, S. Bowhead Whale *Balaena mysticetus* seasonal selection of sea ice. *Mar. Ecol. Prog Ser.* **411**, 285–297 (2010).

[7] George, J. C., Clark, C., Carroll, G. M. & Ellison, W. T. Observations on the ice-breaking and ice navigation behavior of migrating bowhead whales (Balaena mysticetus) near point Barrow, Alaska, spring 1985. *Arctic* **42**, 24–30 (1989).

[8] Vacquié-Garcia, J. et al. Late summer distribution and abundance of ice-associated whales in the Norwegian high Arctic. *Endanger. Species Res.* **32**, 59–70 (2017).

[9] Matthews, C. J. D., Breed, G. A., LeBlanc, B. & Ferguson, S. H. Killer whale presence drives bowhead whale selection for sea ice in Arctic seascapes of fear. *Proc. Natl. Acad. Sci.* 117, 6590–6598 (2020). 10.1073/pnas.1911761117 PMC7104343 32152110

[10] Kovacs, K. M. et al. The endangered Spitsbergen bowhead whales’ secrets revealed after hundreds of years in hiding. *Biol. Lett.* **16**, 20200148 (2020). 32516566 10.1098/rsbl.2020.0148 PMC7336847

[11] Allen, R. C. & Keay, I. Bowhead whales in the Eastern Arctic, 1611–1911: Population reconstruction with historical whaling. *Environ. Hist.* **12**, 89–113 (2006).

[12] Christensen, I., Haug, T. & Øien, N. Seasonal distribution, exploitation and present abundance of stocks of large Baleen whales (*Mysticeti*) and sperm whales (*Physeter macrocephalus*) in Norwegian and adjacent waters. *ICES J. Mar. Sci.* **49**, 341–355 (1992).

[13] Boertmann, D., Kyhn, L. A. & Witting, L. A hidden getaway for bowhead whales in the Greenland sea. *Polar Biol.* **38**, 1315–1319 (2015).

[14] Tervo, O. M., Louis, M., Sinding, M-H-S., Heide-Jørgensen, M. P. & Hansen, R. G. Possible signs of recovery of the nearly extirpated Spitsbergen bowhead whales: Calves observed in East Greenland. *Polar Res.* **42**, 8809 (2023).

[15] Heide-Jørgensen, M. P., Laidre, K. L., Jensen, M. V., Dueck, L. & Postma, L. D. Dissolving stock discreteness with satellite tracking: Bowhead whales in Baffin Bay. *Mar. Mammal Sci.* **22**, 34–45 (2006).

[16] Quakenbush, L. T., Citta, J., George, J., Small, R. J. & Heide-Jørgensen, M. P. Fall and winter movements of bowhead whales (*Balaena mysticetus*) in the Chukchi sea and within a potential petroleum development area. *Arctic* **63**, 289–307 (2010).

[17] Szesciorka, A. R. & Stafford, K. M. Sea ice directs changes in bowhead Whale phenology through the Bering Strait. *Mov. Ecol.* **11**, 8 (2023). 36750903 10.1186/s40462-023-00374-5 PMC9903510

[18] Lydersen, C. et al. Lost highway not forgotten: satellite tracking of a bowhead Whale (*Balaena mysticetus*) from the critically endangered Spitsbergen stock. *Arctic* **65**, 76–86 (2012).

[19] Wiig, Ø., Bachmann, L., Janik, V. M., Kovacs, K. M. & Lydersen, C. Spitsbergen bowhead whales revisited. *Mar. Mammal Sci.* **23**, 688–693 (2007).

[20] Moore, S. E. Variability of cetacean distribution and habitat selection in the Alaskan Arctic, autumn 1982-91. *Arctic* **53**, 448–460 (2000).

[21] Shpak, O. & Paramonov, A. Y. The bowhead whale, *Balaena mysticetus* Linnaeus, 1758, in the Western sea of Okhotsk (2009–2016): distribution pattern, behavior, and threats. *Russ J. Mar. Biol.* **44**, 210–218 (2018).

[22] Citta, J. J. et al. Shifts in bowhead Whale distribution, behavior, and condition following rapid sea ice change in the Bering sea. *Cont. Shelf Res.* **256**, 104959 (2023).

[23] Robbins, J. et al. Satellite tag effectiveness and impacts on large whales: preliminary results of a case study with Gulf of Maine humpback whales. SC/65A/SH05 presented to the IWC Scientific Committee, Korea, (2013).

[24] Charif, R. A. & Clark, C. W. Acoustic monitoring of large whales in deep waters North and West of the British Isles: 1996–2005. *Cornell Lab. Ornithol.* **8**, 40 (2009).

[25] Ahonen, H. et al. The underwater soundscape in Western Fram Strait: Breeding ground of Spitsbergen’s endangered bowhead whales. *Mar. Pollut Bull.* **123**, 97–112 (2017). 28938997 10.1016/j.marpolbul.2017.09.019

[26] Stafford, K. M., Citta, J. J., Okkonen, S. R. & Zhang, J. Bowhead and beluga Whale acoustic detections in the Western Beaufort sea 2008–2018. *PLoS ONE*. **16**, e0253929 (2021). 34181700 10.1371/journal.pone.0253929 PMC8238202

[27] Stafford, K. M. et al. Spitsbergen’s endangered bowhead whales Sing through the Polar night. *Endanger. Species Res.* **18**, 95–103 (2012).

[28] Clark, C. W. & Johnson, J. H. The sounds of the bowhead whale, *Balaena mysticetus*, during the spring migrations of 1979 and 1980. *Can. J. Zool.* **62**, 1436–1441 (1984).

[29] George, J. C. & Thewissen, J. G. M. The bowhead Whale, *Balaena mysticetus*, Biology and Human Interactions. (Academic, (2020).

[30] Stafford, K. M., Lydersen, C., Wiig, Ø. & Kovacs, K. M. Extreme diversity in the songs of Spitsbergen’s bowhead whales. *Biol. Lett.* **14**, 20180056 (2018). 29618521 10.1098/rsbl.2018.0056 PMC5938564

[31] Diogou, N. et al. Bowhead Whale year-round acoustic presence and habitat associations in the Amundsen Gulf, Western Canadian Arctic, 2018–2019. *Prog Oceanogr.* **213**, 103004 (2023).

[32] Chambault, P. et al. Sea surface temperature predicts the movements of an Arctic cetacean: the bowhead Whale. *Sci. Rep.* **8**, 9658 (2018). 29942009 10.1038/s41598-018-27966-1 PMC6018504

[33] Llobet, S. M., Ahonen, H., Lydersen, C. & Kovacs, K. M. The Arctic and the future Arctic? Soundscapes and marine mammal communities on the East and West sides of Svalbard characterized through acoustic data. *Front. Mar. Sci.* **10**, 1–17 (2023).

[34] Hunkins, K. A review of the physical oceanography of Fram Strait. in The Physical Oceanography of Sea Straits (ed Pratt, L. J.) 61–93 (Springer, Dordrecht, (1990).

[35] Lundesgaard, Ø., Sundfjord, A., Lind, S., Nilsen, F. & Renner, A. H. H. Import of Atlantic water and sea ice controls the ocean environment in the Northern Barents sea. *Ocean. Sci.* **18**, 1389–1418 (2022).

[36] Wold, A. et al. Atlantification influences zooplankton communities seasonally in the Northern Barents sea and Arctic ocean. *Prog Oceanogr.* **219**, 103133 (2023).

[37] Wiig, Ø. Seven bowhead whales *(Balaena mysticetus L.*) observed at Franz Josef Land in 1990. *Mar. Mammal Sci.* **7**, 316–319 (2006).

[38] Gavrilo, M. V. *Status of the Bowhead Whale Balaena mysticetus in the Waters of Franz Josef Land Archipelago. SC/66a/BRG/202015 Presented To the IWC* (Scientific Committee, 2015).

[39] Hunt, K. E. et al. Male bowhead Whale reproductive histories inferred from Baleen testosterone and stable isotopes. *Integr. Org. Biol.* **4**, obac014 (2022). 35617113 10.1093/iob/obac014 PMC9125798

[40] de la Castro, L. et al. Assessing net primary production in the Northwestern Barents sea using in situ, remote sensing and modelling approaches. *Prog Oceanogr.* **219**, 103160 (2023).

[41] Pomerleau, C., Lesage, V., Winkler, G., Rosenberg, B. & Ferguson, S. H. Fatty acid biomarkers infer contemporary diet of bowhead whales (*Balaena mysticetus*) from the Eastern Canadian Arctic. *Arctic* **67**, 84–92 (2014).

[42] Pomerleau, C. et al. Mercury and stable isotope cycles in Baleen plates are consistent with year-round feeding in two bowhead Whale (*Balaena mysticetus*) populations. *Polar Biol.* **41**, 1881–1893 (2018).

[43] Citta, J. J. et al. Ecological characteristics of core-use areas used by Bering-Chukchi-Beaufort (BCB) bowhead whales, 2006–2012. *Prog Oceanogr.* **136**, 201–222 (2015).

[44] Moore, S. E. Is it ‘boom times’ for Baleen whales in the Pacific Arctic region? *Biol. Lett.* **12**, 20160251 (2016). 27601724 10.1098/rsbl.2016.0251 PMC5046915

[45] Hirche, H. J. & Kosobokova, K. N. Winter studies on zooplankton in Arctic seas: the Storfjord (Svalbard) and adjacent ice-covered Barents sea. *Mar. Biol.* **158**, 2359–2376 (2011).

[46] Stafford, K. M. Singing behavior in the bowhead Whale. in Ethology and Behavioral Ecology of Mysticetes (eds Clark, C. W. & Garland, E. C.) 277–295 (Springer International Publishing, Cham, (2022).

[47] Gillespie, D. et al. PAMGUARD: semiautomated, open-source software for real-time acoustic detection and localization of cetaceans. *Proc. Inst. Acoust.* **30**, 54–62 (2008).

[48] Spreen, G., Kaleschke, L. & Heygster, G. Sea ice remote sensing using AMSR-E 89-GHz channels. *J. Geophys. Res. : Oceans*. **113**, C2 (2008).

[49] Pebesma, E. & Bivand, R. Classes and methods for Spatial data in R. *R News*. **5**, 9–13 (2005).

[50] Hijmans, R. raster: geographic data analysis and modeling. R package version 3.6–26. (2023). https://cran.r-project.org/package=raster

[51] Bonnel, J., Thode, A. M., Blackwell, S. B., Kim, K. & Macrander, A. M. Range Estimation of bowhead Whale (*Balaena mysticetus*) calls in the Arctic using a single hydrophone. *J. Acoust. Soc. Am.* **136**, 145–155 (2014). 24993202 10.1121/1.4883358

[52] Fetterer, F., Meier, W., Savoie, M. & Windnagel, A. *Sea Ice Index: Ice Cover and Trends in Ice Cover in the Arctic and Antarctic Oceans, Version 3, 1978-present* (National Snow and Ice Data Center, 2008).

[53] Bivand, R., Keitt, T. & Rowlingson, B. rgdal: bindings for the ‘geospatial’ data abstraction library. R package version 1.6.7. (2023). https://cran.r-project.org/package=rgdal

[54] Global ocean biogeochemistry hindcast. E.U. Copernicus Marine Service Information (CMEMS). Marine Data Store (MDS).

[55] Global ocean low and mid trophic levels biomass content hindcast. E.U. Copernicus marine service information (CMEMS). Marine Data Store (MDS).

[56] Good, S. et al. The current configuration of the OSTIA system for operational production of foundation sea surface temperature and ice concentration analyses. *Remote Sens.* **12**, 720. 10.3390/rs12040720 (2020).

[57] Wood, S. N. Fast stable restricted maximum likelihood and marginal likelihood Estimation of semiparametric generalized linear models. *J. R Stat. Soc. B: Stat. Methodol.* **73**, 3–36 (2011).

[58] Fox, J. & Weisberg, S. An R companion to applied regression, third edition. Sage, Thousand Oaks, CA (2019).

[59] Pinheiro, J. & Bates, D. nlme: linear and nonlinear mixed effects models. R package version 3.1.164. (2024). https://CRAN.R-project.org/package=nlme

[60] Tweedie, M. C. K. An index which distinguishes between some important exponential families. in *Statistics: applications and new directions* (ed. Ghosh, J. K. & Roy, J.) 579–604Indian Statistical Institute, Calcutta, (1984).

[61] Wood, S. N., Pya, N. & Säfken, B. Smoothing parameter and model selection for general smooth models. *J. Am. Stat. Assoc.* **111**, 1548–1563 (2016).

[62] Zuur, A. F., Iena, E. N., Walker, N. J., Saveliev, A. A. & Smith, G. M. GLM and GAM for absence–presence and proportional data. in Mixed Effects Models and Extensions in Ecology with R (eds Zuur, A. F., Ieno, E. N., Walker, N., Saveliev, A. A. & Smith, G. M.) 245–259 (Springer-, New York, (2009).

[63] Wickham, H. ggplot2: elegant graphics for data analysis. Springer-Verlag, New York. ISBN 978-3-319-24277-4. https://ggplot2.tidyverse.org

