# Supplementary material for: Singers of the high Arctic; Seasonal acoustic presence of bowhead whales (Balaena mysticetus) around Svalbard, Norway
Source: Sci Rep. 2025 Apr 25;15:14425. doi: 10.1038/s41598-025-98302-7 (PMC12032261; doi:10.1038/s41598-025-98302-7)
Supplement: Supplementary file 1 — Supplementary Material 1 [file 41598_2025_98302_MOESM1_ESM.docx]

Singers of the High Arctic; Seasonal acoustic presence of bowhead whales (*Balaena mysticetus*) around Svalbard, Norway

Luca Wams^1^, Kit M. Kovacs^1^, Christian Lydersen^1^, Ulf Lindstrøm^2,3^, Dag Tollefsen^4^, Heidi Ahonen^1^

**Manuscript Appendix A**

**
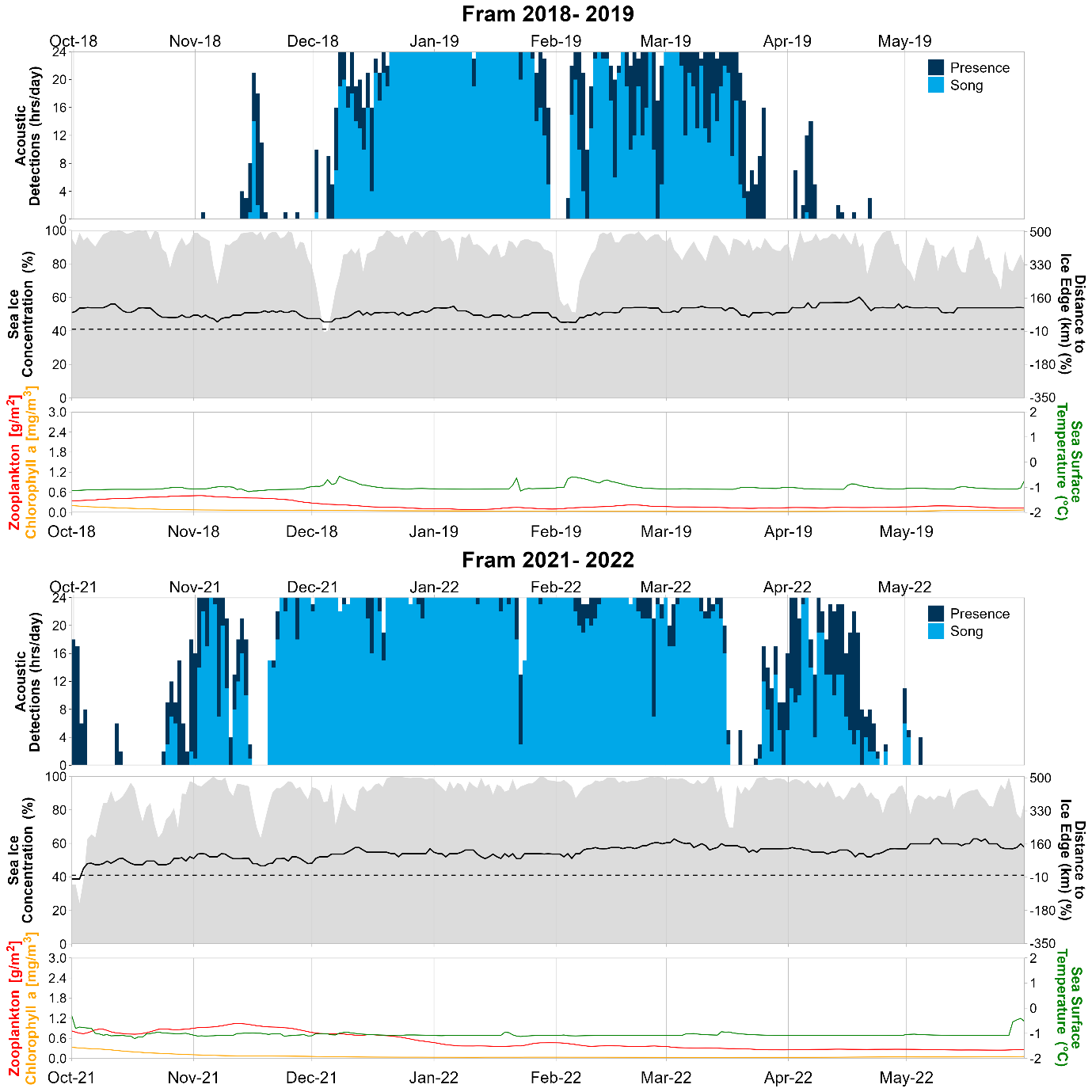
**

***b***

***a***

**Figure A1.** Bowhead whale acoustic presence in relation to environmental variables at Fram Strait in 2018-2019 **(a)** and 2021-2022 **(b)**. Top panel: bowhead whale daily acoustic presence (hrs/day) divided into total presence and song presence. Middle panel: sea ice concentration (%) (grey shaded area) and distance to ice edge (km). The dotted line represents mooring position relative to the ice edge, with negative distance indicating that the mooring is in open water and positive distance indicating ice-cover. Bottom panel: zooplankton (g/m^2^) (red line) and chlorophyll a (mg/m^3^) (yellow line) concentrations and sea surface temperature (°C) (green line).


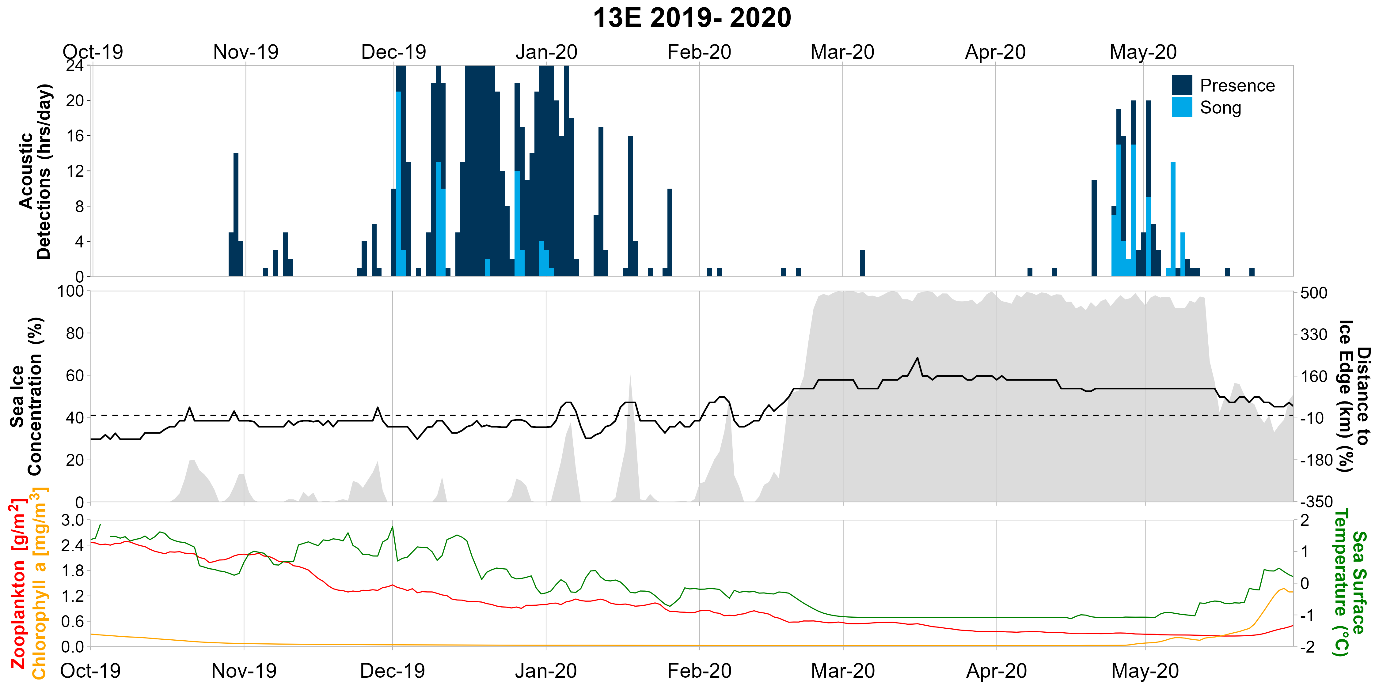


**Figure A2.** Bowhead whale acoustic presence in relation to environmental variables at 13E in 2019-2020. Top panel: bowhead whale daily acoustic presence (hrs/day) divided into total presence and song presence. Middle panel: sea ice concentration (%) (grey shaded ‘area) and distance to ice edge (km). The dotted line represents mooring position relative to the ice edge, with negative distance indicating that the mooring is in open water and positive distance indicating ice-cover. Bottom panel: zooplankton (g/m^2^) (red line) and chlorophyll a (mg/m^3^) (yellow line) concentrations and sea surface temperature (°C) (green line).


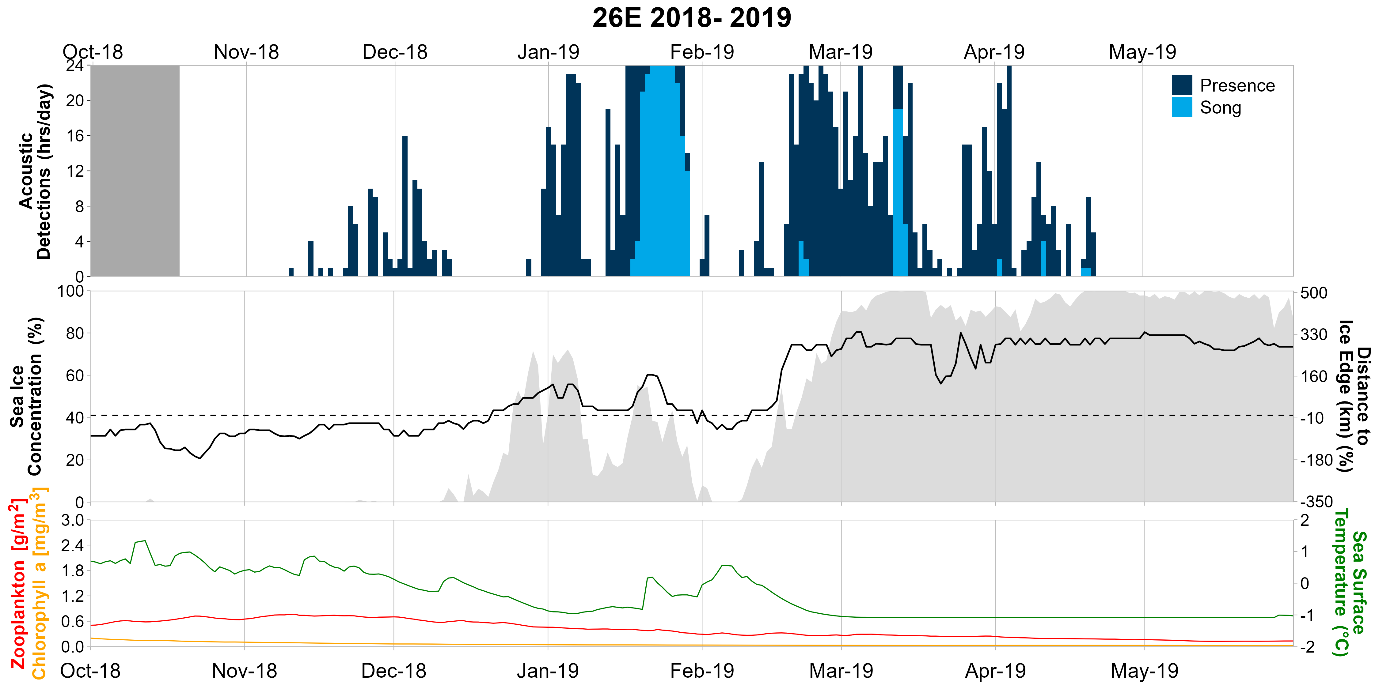


**Figure A3.** Bowhead whale acoustic presence in relation to environmental variables at 26E in 2018-2019. Top panel: bowhead whale daily acoustic presence (hrs/day) divided into total presence and song presence. The grey area indicates days without acoustic data. Middle panel: sea ice concentration (%) (grey shaded area) and distance to ice edge (km). The dotted line represents mooring position relative to the ice edge, with negative distance indicating that the mooring is in open water and positive distance indicating ice-cover. Bottom panel: zooplankton (g/m^2^) (red line) and chlorophyll a (mg/m^3^) (yellow line) concentrations and sea surface temperature (°C) (green line).


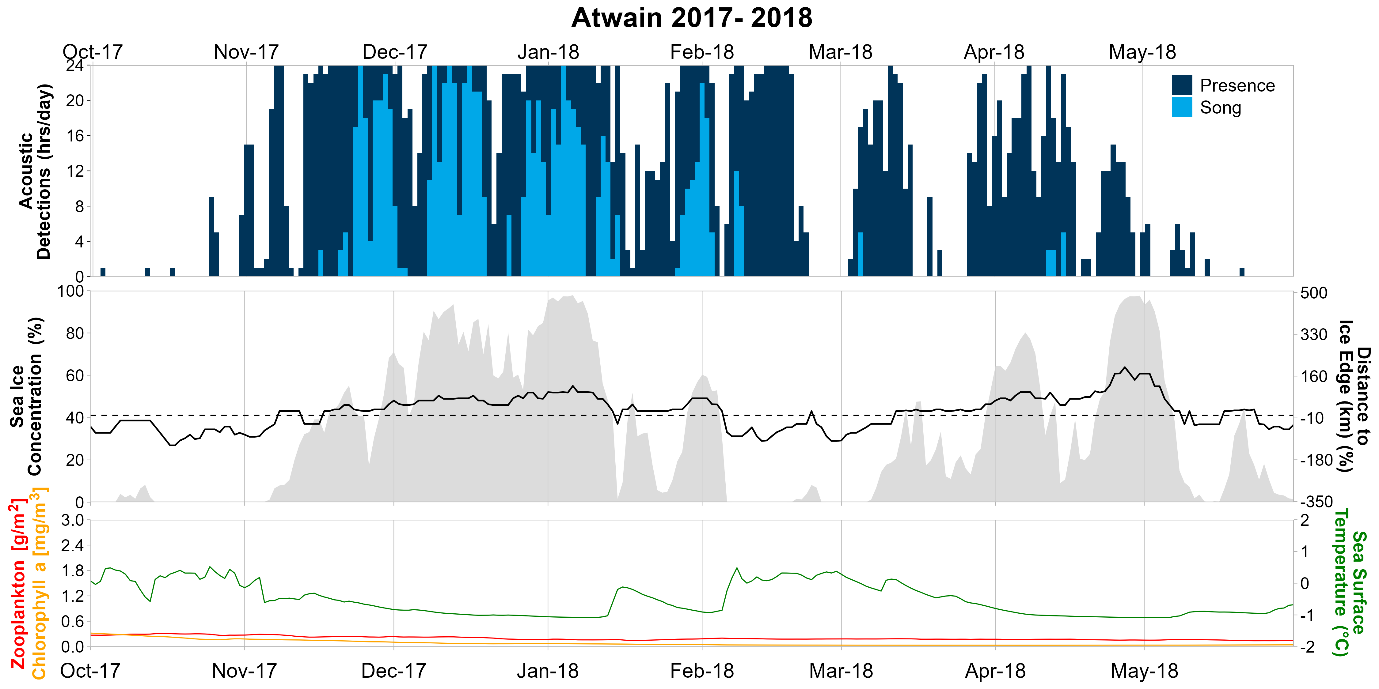


**Figure A4.** Bowhead whale acoustic presence in relation to environmental variables at Atwain in 2017-2018. Top panel: bowhead whale daily acoustic presence (hrs/day) divided into total presence and song presence. Middle panel: sea ice concentration (%) (grey shaded area) and distance to ice edge (km). The dotted line represents mooring position relative to the ice edge, with negative distance indicating that the mooring is in open water and positive distance indicating ice-cover. Bottom panel: zooplankton (g/m^2^) (red line) and chlorophyll a (mg/m^3^) (yellow line) concentrations and sea surface temperature (°C) (green line).


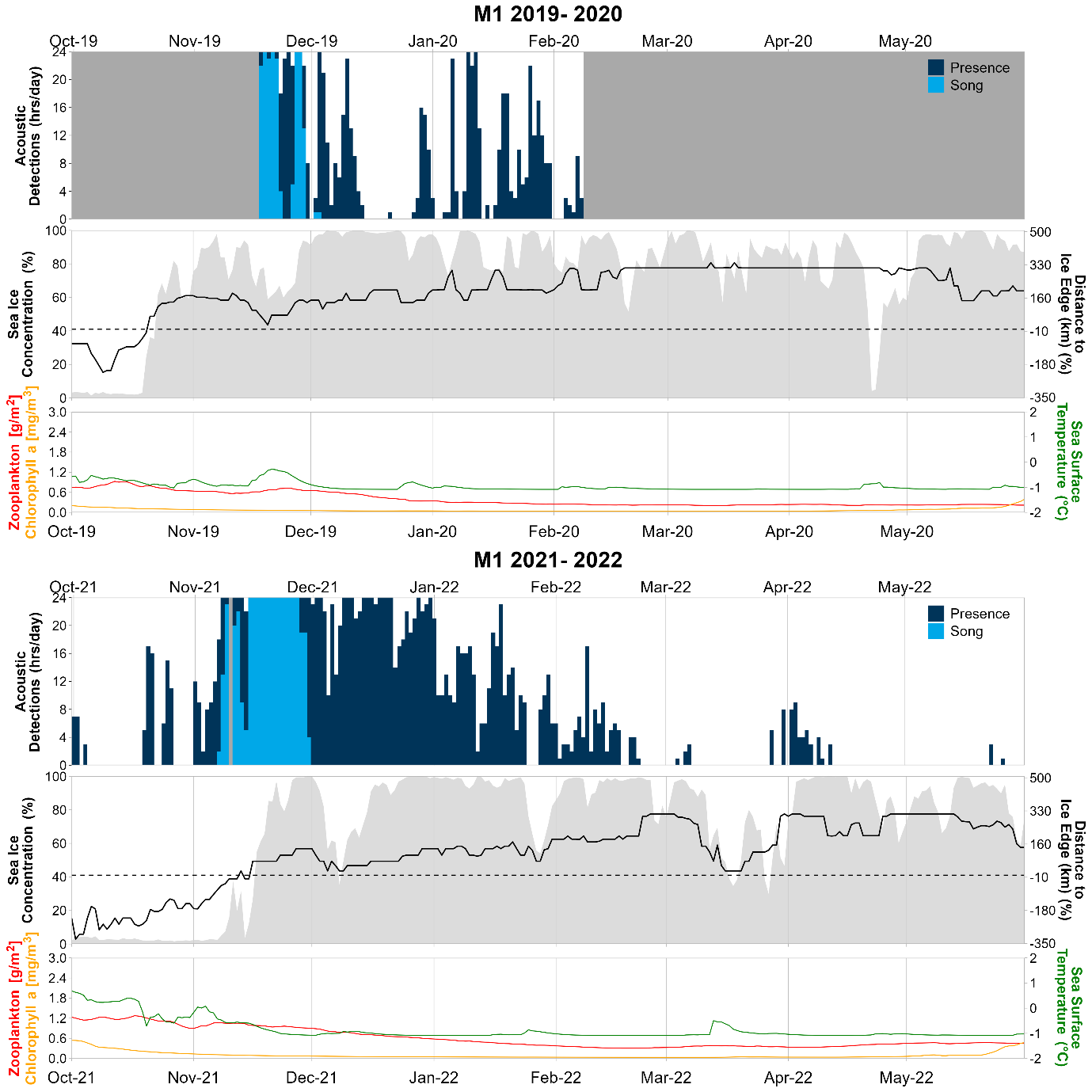

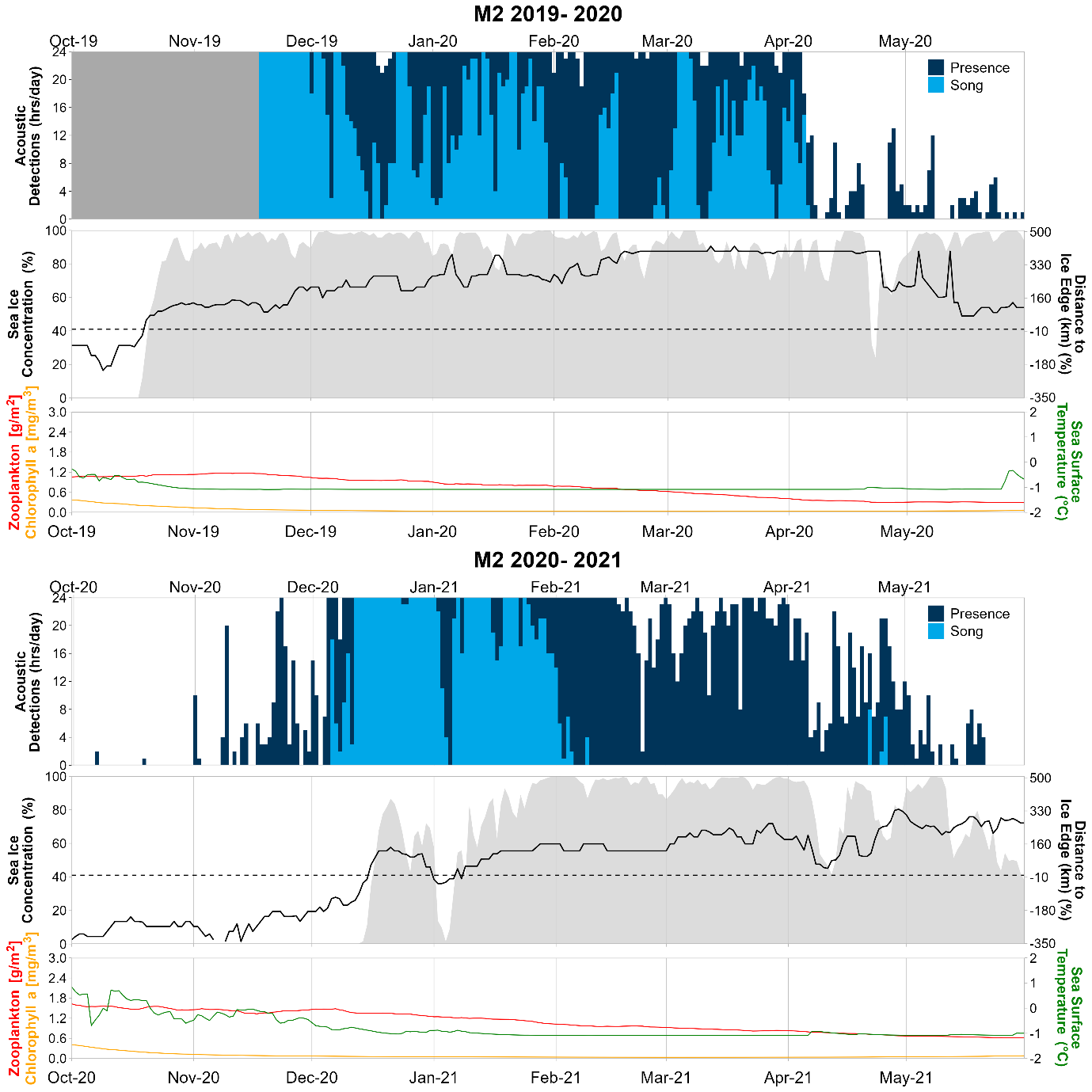


**Figure A5.** Bowhead whale acoustic presence in relation to environmental variables at M1 in **(a)** 2019-2020 and **(b)** 2021-2022. Top panel: bowhead whale daily acoustic presence (hrs/day) divided into total presence and song presence. The grey area indicates days without acoustic data. Middle panel: sea ice concentration (%) (grey shaded area) and distance to ice edge (km). The dotted line represents mooring position relative to the ice edge, with negative distance indicating that the mooring is in open water and positive distance indicating ice-cover. Bottom panel: zooplankton (g/m^2^) (red line) and chlorophyll a (mg/m^3^) (yellow line) concentrations and sea surface temperature (°C) (green line).

***b***

***a***

**Figure A6.** Bowhead whale acoustic presence in relation to environmental variables at M2 in **(a)** 2019-2020 and **(b)** 2020-2021. Top panel: bowhead whale daily acoustic presence (hrs/day) divided into total presence and song presence. The grey area indicates days without acoustic data. Middle panel: sea ice concentration (%) (grey shaded area) and distance to ice edge (km). The dotted line represents mooring position relative to the ice edge, with negative distance indicating that the mooring is in open water and positive distance indicating ice-cover. Bottom panel: zooplankton (g/m^2^) (red line) and chlorophyll a (mg/m^3^) (yellow line) concentrations and sea surface temperature (°C) (green line).

***b***

***a***

**Manuscript Appendix B**

***Table B1.*** *Model output for effects of Sea Ice Concentration (SIC) and month on acoustic presence at Fram Strait (n=484).*

| **Family** | | | **Link Function** | **Formula** | | | **Adjusted R^2^** | | | | **Deviance Explained** | | | | |
| --- | --- | --- | --- | --- | --- | --- | --- | --- | --- | --- | --- | --- | --- | --- | --- |
| Quasibinomial | | | Logit | Presence ~ s(SIC, by = sampling period,  k = 3) + s(month, by = sampling period,  k = 3) + sampling period | | | 0.725 | | | | 66.5% | | | | |
|  |  | **Parametric Coeffficients** | | | | | | | | | | |  | |  |
|  | | | | **Estimate** | **Std. Error** | **T value** | | | | **Pr(>\|t\|)** | | | | | |
| (Intercept) | | | | -2.3242 | 0.2670 | -8.704 | | | | < 2e-16 ^***^ | | | | | |
| SamplingPeriodFramStrait21-22 | | | | 2.1388 | 0.3128 | 6.838 | | | | 2.48e-11 ^***^ | | | | | |
|  |  | **Approximate Significance of Smooth Terms** | | | | | | | | | | |  | |  |
|  | | | | **edf** | **Ref.df.** | **F** | | | | **p-value** | | | | | |
| s(SIC):SamplingPeriod18-19 | | | | 1.233 | 1.411 | 22.70 | | | | 8.08e-07 ^***^ | | | | | |
| s(SIC):SamplingPeriod21-22 | | | | 1.966 | 1.999 | 13.28 | | | | 3.84e-06 ^***^ | | | | | |
| s(month):SamplingPeriod18-19 | | | | 1.994 | 2.000 | 56.70 | | | | < 2e-16 ^***^ | | | | | |
| s(month):SamplingPeriod21-22 | | | | 1.991 | 2.000 | 46.60 | | | | < 2e-16 ^***^ | | | | | |
| **Signif.codes: 0 ‘^***^’ 0.001 ‘^**^’ 0.01 ‘^*^’ 0.05 ‘^.^’ 0.1‘’ 1** | | | | | | | |  |  | | |  | |  | |

***Table B2.*** *Model output for effects of Sea Ice Concentration (SIC) and month on song presence at Fram Strait (n=484).*

| **Family** | | | **Link Function** | **Formula** | | | **Adjusted R^2^** | | | | **Deviance Explained** | | | | |
| --- | --- | --- | --- | --- | --- | --- | --- | --- | --- | --- | --- | --- | --- | --- | --- |
| Quasibinomial | | | Logit | Song ~ s(SIC, by = sampling period,  k = 3) + s(month, by = sampling period,  k = 3) + sampling period | | | 0.692 | | | | 64.8% | | | | |
|  |  | **Parametric Coeffficients** | | | | | | | | | | |  | |  |
|  | | | | **Estimate** | **Std. Error** | **T value** | | | | **Pr(>\|t\|)** | | | | | |
| (Intercept) | | | | -1.4554 | 0.2279 | -6.385 | | | | 4.08e-10 ^***^ | | | | | |
| SamplingPeriodFramStrait21-22 | | | | 2.2806 | 0.2700 | 8.446 | | | | 3.70e-16 ^***^ | | | | | |
|  |  | **Approximate Significance of Smooth Terms** | | | | | | | | | | |  | |  |
|  | | | | **edf** | **Ref.df.** | **F** | | | | **p-value** | | | | | |
| s(SIC):SamplingPeriod18-19 | | | | 1.000 | 1.000 | 26.321 | | | | 1.11e-06 ^***^ | | | | | |
| s(SIC): SamplingPeriod21-22 | | | | 1.642 | 1.872 | 8.106 | | | | 0.00111 ^**^ | | | | | |
| s(month): SamplingPeriod18-19 | | | | 1.993 | 2.000 | 52.353 | | | | < 2e-16 ^***^ | | | | | |
| s(month): SamplingPeriod21-22 | | | | 1.993 | 2.000 | 55.399 | | | | < 2e-16 ^***^ | | | | | |
| **Signif.codes: 0 ‘^***^’ 0.001 ‘^**^’ 0.01 ‘^*^’ 0.05 ‘^.^’ 0.1‘’ 1** | | | | | | | |  |  | | |  | |  | |

***
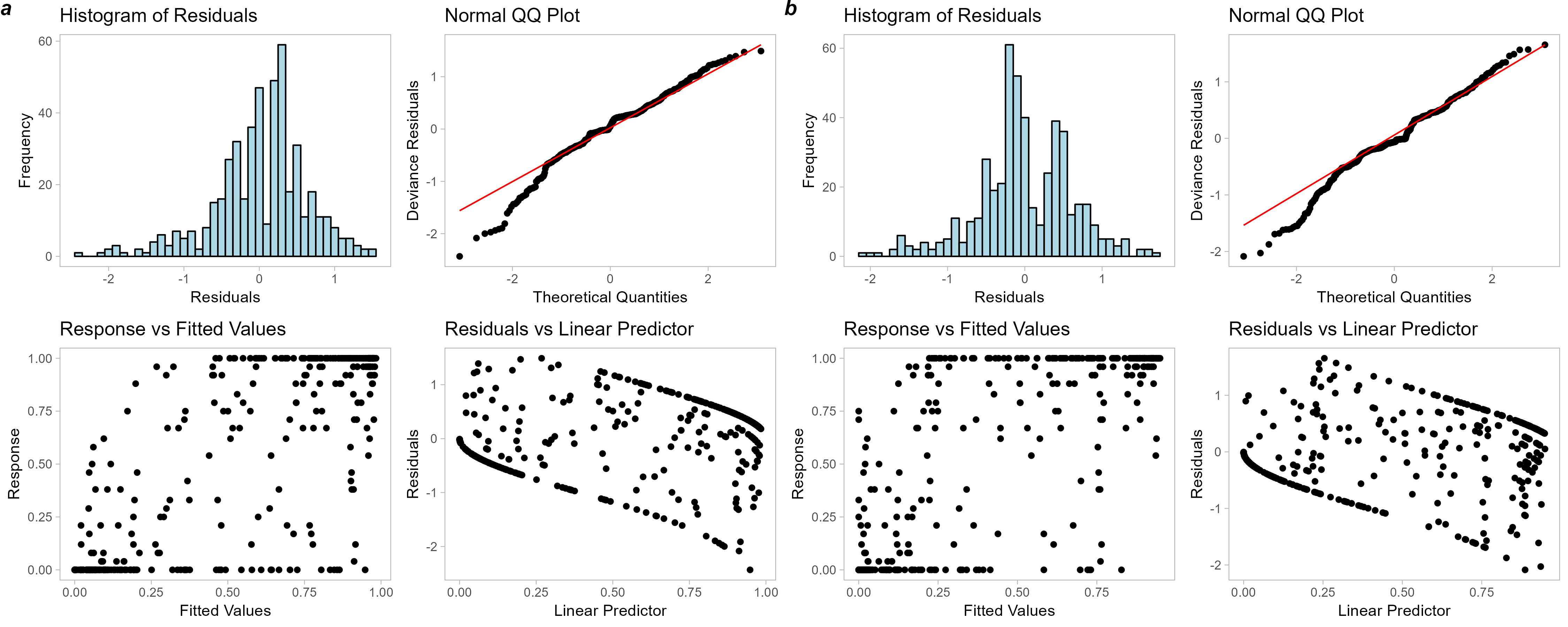
Figure B1.*** *Residual plots for GAMs (Generalized Additive Models) of* ***(a)*** *acoustic presence and* ***(b)*** *song presence of bowhead whales at Fram Strait (n=484). Showing Histogram of Residuals, Q-Q plot, Response vs Fitted Values and Residuals vs Linear Predictor.*

***b***

***a***

***Table B3.*** *Model output for effects of Sea Ice Concentration (SIC) and month on acoustic presence at 13E (n=244).*

| **Family** | | | **Link Function** | **Formula** | | | **Adjusted R^2^** | | | | **Deviance Explained** | | | | |
| --- | --- | --- | --- | --- | --- | --- | --- | --- | --- | --- | --- | --- | --- | --- | --- |
| Quasibinomial | | | Logit | Presence ~ s(SIC, k = 3) +  s(month, k = 3) | | | 0.0904 | | | | 10.5% | | | | |
|  |  | **Parametric Coeffficients** | | | | | | | | | | |  | |  |
|  | | | | **Estimate** | **Std. Error** | **T value** | | | | **Pr(>\|t\|)** | | | | | |
| (Intercept) | | | | -1.9740 | 0.1692 | -11.66 | | | | < 2e-16 ^***^ | | | | | |
|  |  | **Approximate Significance of Smooth Terms** | | | | | | | | | | |  | |  |
|  | | | | **edf** | **Ref.df.** | **F** | | | | **p-value** | | | | | |
| s(SIC) | | | | 1.039 | 1.077 | 4.237 | | | | 0.03918 ^*^ | | | | | |
| s(month) | | | | 1.910 | 1.992 | 4.833 | | | | 0.00952 ^**^ | | | | | |
| **Signif.codes: 0 ‘^***^’ 0.001 ‘^**^’ 0.01 ‘^*^’ 0.05 ‘^.^’ 0.1‘’ 1** | | | | | | | |  |  | | |  | |  | |

***Table B4.*** *Model output for effects of Sea Ice Concentration (SIC) and month on song presence at 13E (n=244).*

| **Family** | | | **Link Function** | **Formula** | | | **Adjusted R^2^** | | | | **Deviance Explained** | | | | |
| --- | --- | --- | --- | --- | --- | --- | --- | --- | --- | --- | --- | --- | --- | --- | --- |
| Quasibinomial | | | Logit | Song ~ s(SIC, k = 3) +  s(month, k = 3) | | | 0.0062 | | | | 5.26% | | | | |
|  |  | **Parametric Coeffficients** | | | | | | | | | | |  | |  |
|  | | | | **Estimate** | **Std. Error** | **T value** | | | | **Pr(>\|t\|)** | | | | | |
| (Intercept) | | | | -3.8620 | 0.3396 | -11.37 | | | | < 2e-16 ^***^ | | | | | |
|  |  | **Approximate Significance of Smooth Terms** | | | | | | | | | | |  | |  |
|  | | | | **edf** | **Ref.df.** | **F** | | | | **p-value** | | | | | |
| s(SIC) | | | | 1.887 | 1.987 | 1.157 | | | | 0.344 | | | | | |
| s(month) | | | | 1.000 | 1.000 | 1.861 | | | | 0.174 | | | | | |
| **Signif.codes: 0 ‘^***^’ 0.001 ‘^**^’ 0.01 ‘^*^’ 0.05 ‘^.^’ 0.1‘’ 1** | | | | | | | |  |  | | |  | |  | |

***
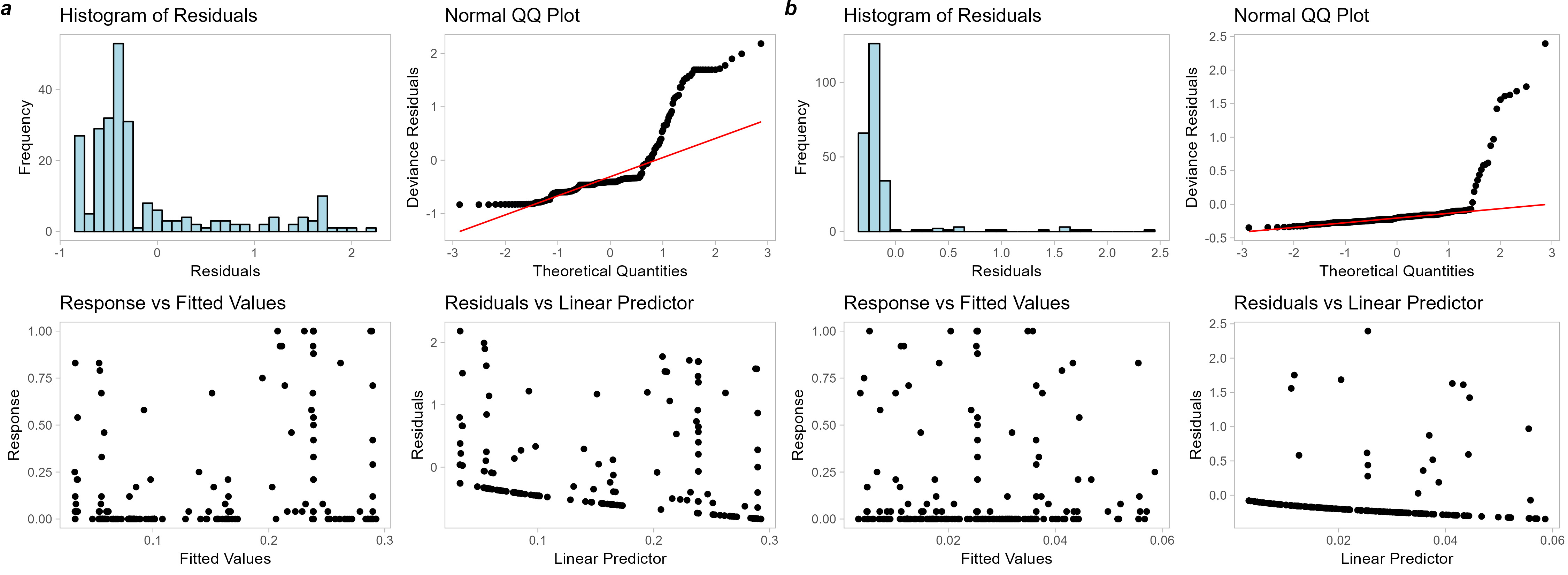

Figure B2.*** *Residual plots for GAMs (Generalized Additive Models) of* ***(a)*** *acoustic presence and* ***(b)*** *song presence of bowhead whales at 13E (n=244). Showing Histogram of Residuals, Q-Q plot, Response vs Fitted Values and Residuals vs Linear Predictor.*

***b***

***a***

***Table B5.*** *Model output for effects of Sea Ice Concentration (SIC) and month on acoustic presence at 26E (n=225).*

| **Family** | | | **Link Function** | **Formula** | | | **Adjusted R^2^** | | | | **Deviance Explained** | | | | |
| --- | --- | --- | --- | --- | --- | --- | --- | --- | --- | --- | --- | --- | --- | --- | --- |
| Quasibinomial | | | Logit | Presence ~ s(SIC, k = 3) +  s(month, k = 3) | | | 0.457 | | | | 43.4% | | | | |
|  |  | **Parametric Coeffficients** | | | | | | | | | | |  | |  |
|  | | | | **Estimate** | **Std. Error** | **T value** | | | | **Pr(>\|t\|)** | | | | | |
| (Intercept) | | | | -1.81971 | 0.18184 | -10.007 | | | | < 2.2e-16 ^***^ | | | | | |
|  |  | **Approximate Significance of Smooth Terms** | | | | | | | | | | |  | |  |
|  | | | | **edf** | **Ref.df.** | **F** | | | | **p-value** | | | | | |
| s(SIC) | | | | 1.8795 | 1.9849 | 16.667 | | | | < 2.39e-07 ^***^ | | | | | |
| s(month) | | | | 1.9762 | 1.9989 | 20.880 | | | | < 2.2e-16 ^***^ | | | | | |
| **Signif.codes: 0 ‘^***^’ 0.001 ‘^**^’ 0.01 ‘^*^’ 0.05 ‘^.^’ 0.1‘’ 1** | | | | | | | |  |  | | |  | |  | |

***Table B6.*** *Model output for effects of Sea Ice Concentration (SIC) and month on song presence at 26E (n=225).*

| **Family** | | | **Link Function** | **Formula** | | | **Adjusted R^2^** | | | | **Deviance Explained** | | | | |
| --- | --- | --- | --- | --- | --- | --- | --- | --- | --- | --- | --- | --- | --- | --- | --- |
| Quasibinomial | | | Logit | Song ~ s(SIC, k = 3) +  s(month, k = 3) | | | 0.141 | | | | 29.5% | | | | |
|  |  | **Parametric Coeffficients** | | | | | | | | | | |  | |  |
|  | | | | **Estimate** | **Std. Error** | **T value** | | | | **Pr(>\|t\|)** | | | | | |
| (Intercept) | | | | -4.7447 | 0.7528 | -6.3028 | | | | 1.574e-09 ^***^ | | | | | |
|  |  | **Approximate Significance of Smooth Terms** | | | | | | | | | | |  | |  |
|  | | | | **edf** | **Ref.df.** | **F** | | | | **p-value** | | | | | |
| s(SIC) | | | | 1.8143 | 1.9612 | 4.3374 | | | | 0.02220 ^*^ | | | | | |
| s(month) | | | | 1.9312 | 1.9909 | 3.3360 | | | | 0.04081 ^*^ | | | | | |
| **Signif.codes: 0 ‘^***^’ 0.001 ‘^**^’ 0.01 ‘^*^’ 0.05 ‘^.^’ 0.1‘’ 1**  **d** | | | | | | | |  |  | | |  | |  | |


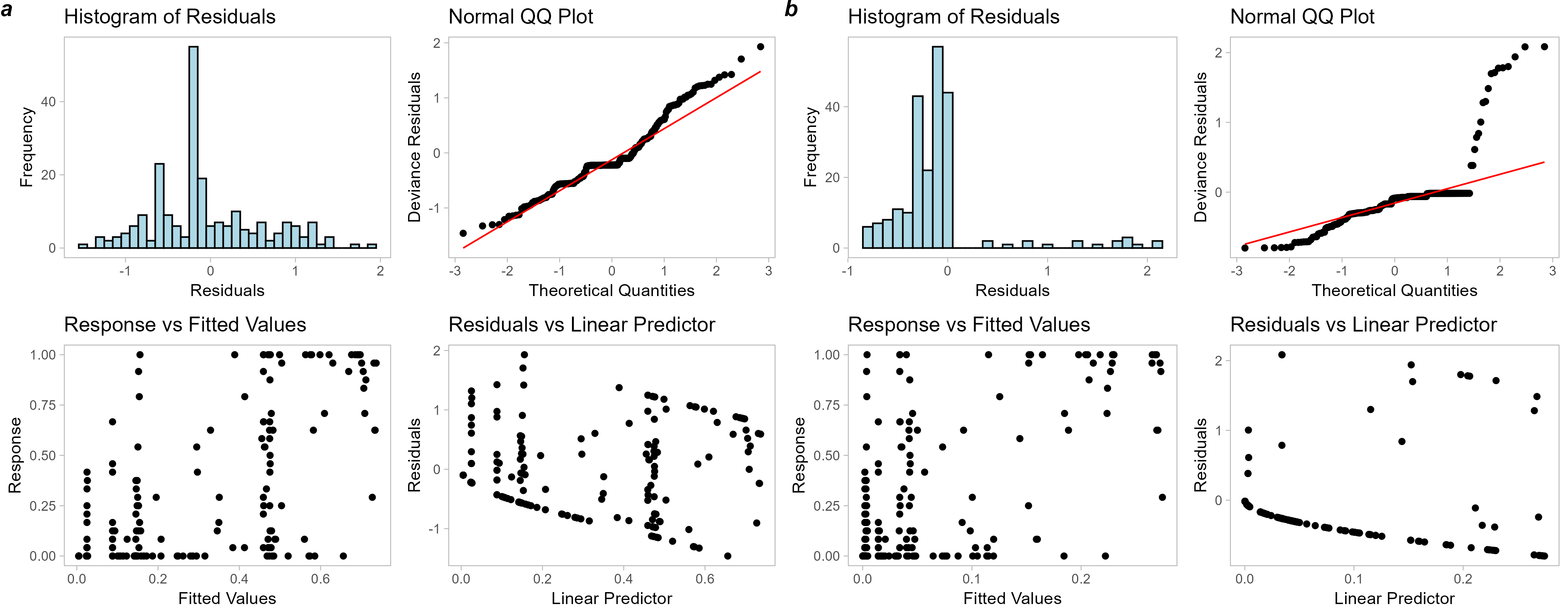


***b***

***a***

***Fig B3.*** *Residual plots for GAMs (Generalized Additive Models) of* ***(a)*** *acoustic presence and* ***(b)*** *song presence of bowhead whales at 26E (n=225). Showing Histogram of Residuals, Q-Q plot, Response vs Fitted Values and Residuals vs Linear Predictor.*

***Table B7.*** *Model output for effects of Sea Ice Concentration (SIC) and month on acoustic presence at Atwain (n=243).*

| **Family** | | | **Link Function** | **Formula** | | | **Adjusted R^2^** | | | | **Deviance Explained** | | | | |
| --- | --- | --- | --- | --- | --- | --- | --- | --- | --- | --- | --- | --- | --- | --- | --- |
| Quasibinomial | | | Logit | Presence ~ s(SIC, k = 3) +  s(month, k = 3) | | | 0.474 | | | | 42.9% | | | | |
|  |  | **Parametric Coeffficients** | | | | | | | | | | |  | |  |
|  | | | | **Estimate** | **Std. Error** | **T value** | | | | **Pr(>\|t\|)** | | | | | |
| (Intercept) | | | | -0.02915 | 0.11914 | -0.245 | | | | 0.807 | | | | | |
|  |  | **Approximate Significance of Smooth Terms** | | | | | | | | | | |  | |  |
|  | | | | **edf** | **Ref.df.** | **F** | | | | **p-value** | | | | | |
| s(SIC) | | | | 1.000 | 1 | 50.96 | | | | < 2e-16 ^***^ | | | | | |
| s(month) | | | | 1.986 | 2 | 38.20 | | | | < 2e-16 ^***^ | | | | | |
| **Signif.codes: 0 ‘^***^’ 0.001 ‘^**^’ 0.01 ‘^*^’ 0.05 ‘^.^’ 0.1‘’ 1** | | | | | | | |  |  | | |  | |  | |

***Table B8.*** *Model output for effects of Sea Ice Concentration (SIC) and month on song presence at Atwain (n=243).*

| **Family** | | | **Link Function** | **Formula** | | | **Adjusted R^2^** | | | | **Deviance Explained** | | | | |
| --- | --- | --- | --- | --- | --- | --- | --- | --- | --- | --- | --- | --- | --- | --- | --- |
| Quasibinomial | | | Logit | Song ~ s(SIC, k = 3) +  s(month, k = 3) | | | 0.508 | | | | 53.1% | | | | |
|  |  | **Parametric Coeffficients** | | | | | | | | | | |  | |  |
|  | | | | **Estimate** | **Std. Error** | **T value** | | | | **Pr(>\|t\|)** | | | | | |
| (Intercept) | | | | -3.3177 | 0.2782 | -11.93 | | | | < 2e-16 ^***^ | | | | | |
|  |  | **Approximate Significance of Smooth Terms** | | | | | | | | | | |  | |  |
|  | | | | **edf** | **Ref.df.** | **F** | | | | **p-value** | | | | | |
| s(SIC) | | | | 1.000 | 1.000 | 63.05 | | | | < 2e-16 ^***^ | | | | | |
| s(month) | | | | 1.955 | 1.998 | 16.59 | | | | 4.06e-07 ^***^ | | | | | |
| **Signif.codes: 0 ‘^***^’ 0.001 ‘^**^’ 0.01 ‘^*^’ 0.05 ‘^.^’ 0.1‘’ 1**  **d** 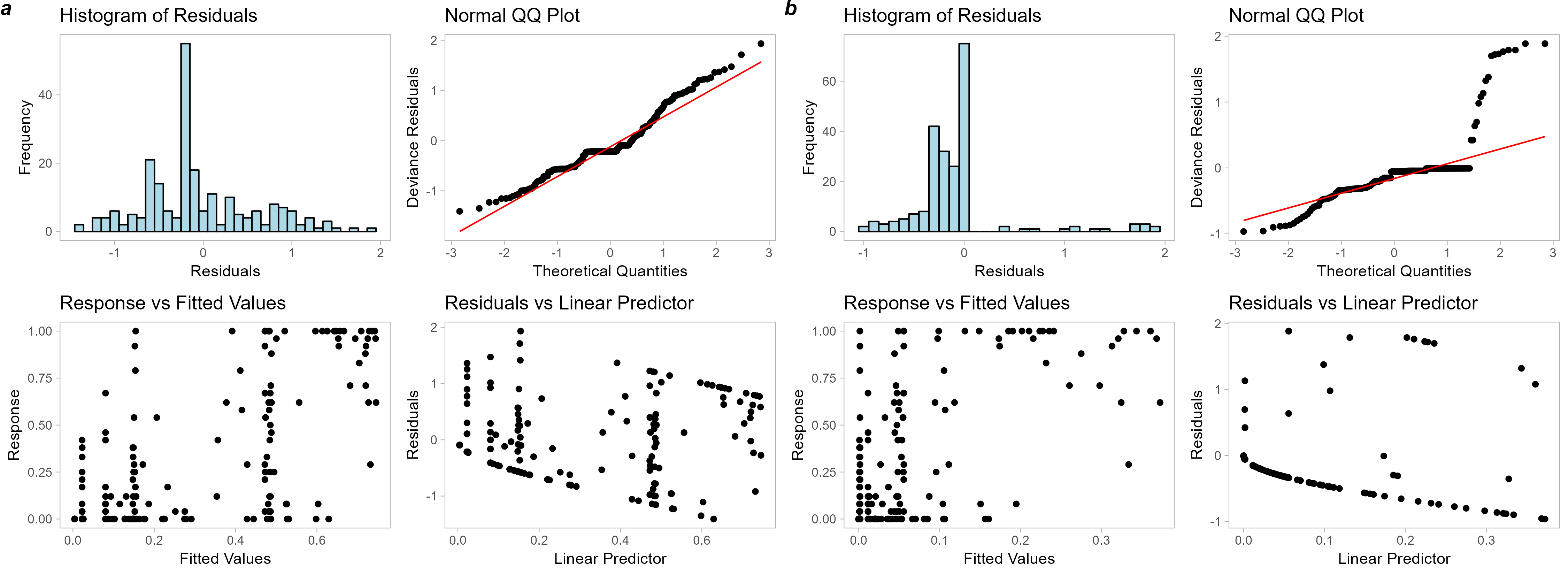 | | | | | | | |  |  | | |  | |  | |

***b***

***a***


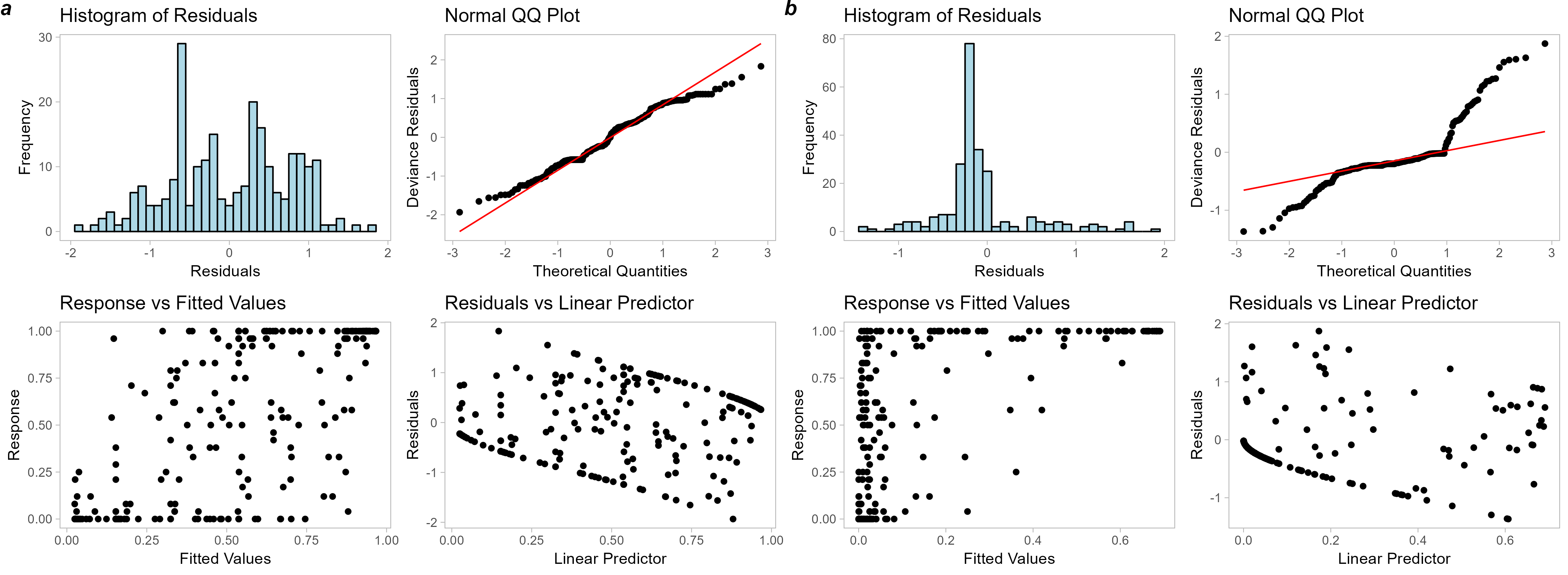


***Figure B4.*** *Residual plots for GAMs (Generalized Additive Models) of* ***(a)*** *acoustic presence and* ***(b)*** *song presence of bowhead whales at Atwain (n=243). Showing Histogram of Residuals, Q-Q plot, Response vs Fitted Values and Residuals vs Linear Predictor.*

***Table B9.*** *Model output for effects of Sea Ice Concentration (SIC) and month on acoustic presence at M1 (n=240).*

| **Family** | | | **Link Function** | **Formula** | | | **Adjusted R^2^** | | | | **Deviance Explained** | | | | |
| --- | --- | --- | --- | --- | --- | --- | --- | --- | --- | --- | --- | --- | --- | --- | --- |
| Quasibinomial | | | Logit | Presence ~ s(SIC, k = 3) +  s(month, k = 3) | | | 0.688 | | | | 60.8% | | | | |
|  |  | **Parametric Coeffficients** | | | | | | | | | | |  | |  |
|  | | | | **Estimate** | **Std. Error** | **T value** | | | | **Pr(>\|t\|)** | | | | | |
| (Intercept) | | | | -1.6930 | 0.1942 | -8.717 | | | | 5.22e-16 ^***^ | | | | | |
|  |  | **Approximate Significance of Smooth Terms** | | | | | | | | | | |  | |  |
|  | | | | **edf** | **Ref.df.** | **F** | | | | **p-value** | | | | | |
| s(SIC) | | | | 1.963 | 1.997 | 36.64 | | | | <2e-16 ^***^ | | | | | |
| s(month) | | | | 1.686 | 1.900 | 65.38 | | | | <2e-16 ^***^ | | | | | |
| **Signif.codes: 0 ‘^***^’ 0.001 ‘^**^’ 0.01 ‘^*^’ 0.05 ‘^.^’ 0.1‘’ 1** | | | | | | | |  |  | | |  | |  | |


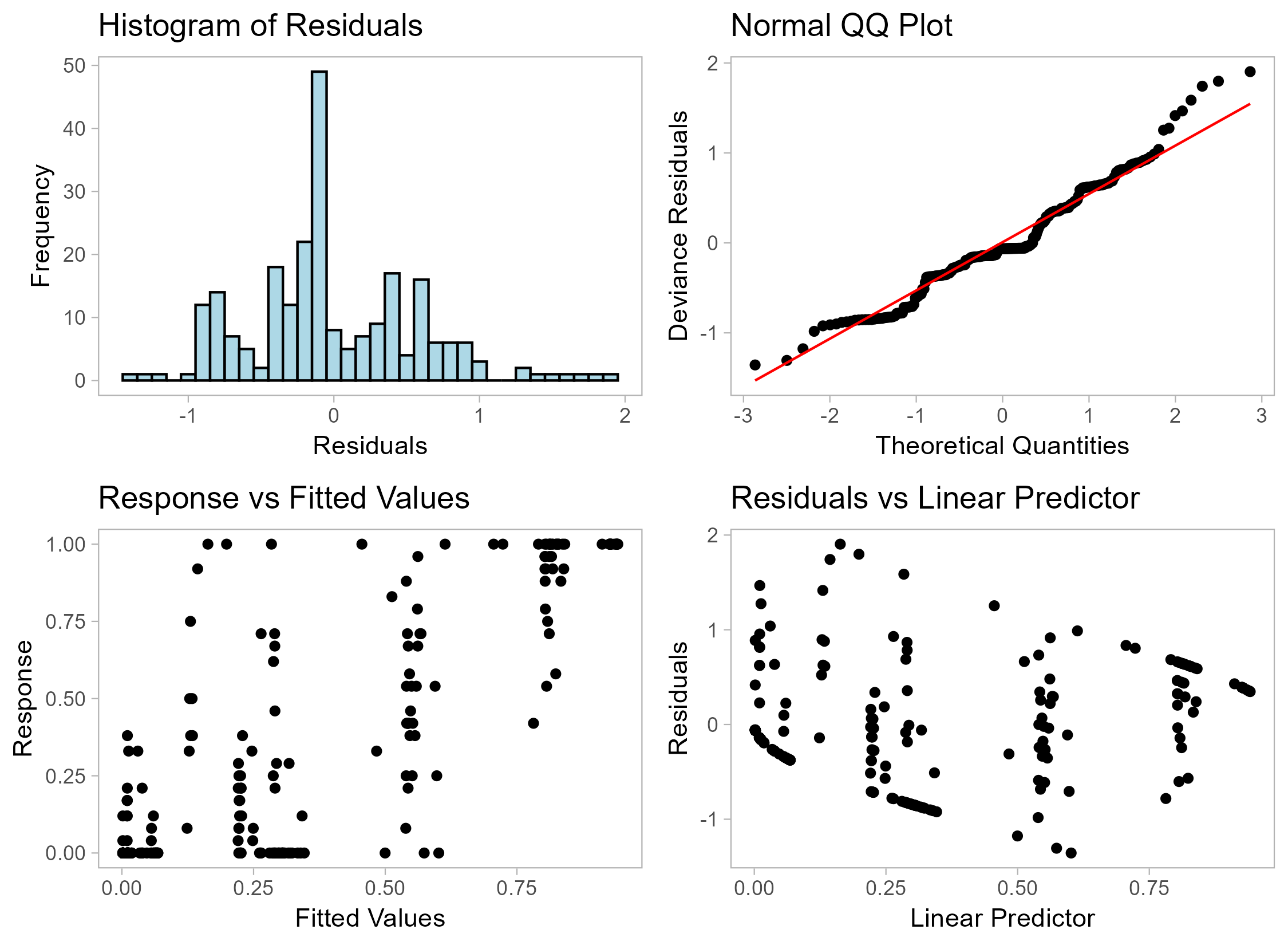


***Figure B5.*** *Residual plots for GAMs (Generalized Additive Models) of acoustic presence of bowhead whales at M1 (n=240). Showing Histogram of Residuals, Q-Q plot, Response vs Fitted Values and Residuals vs Linear Predictor.*

***Table B10.*** *Model output for effects of Sea Ice Concentration (SIC) and month on acoustic presence at M2 (n=439).*

| **Family** | | | **Link Function** | **Formula** | | | **Adjusted R^2^** | | | | **Deviance Explained** | | | | |
| --- | --- | --- | --- | --- | --- | --- | --- | --- | --- | --- | --- | --- | --- | --- | --- |
| Quasibinomial | | | Logit | Presence ~ s(SIC, by = sampling period,  k = 3) + s(month, by = sampling period,  k = 3) + sampling period | | | 0.828 | | | | 77.1% | | | | |
|  |  | **Parametric Coeffficients** | | | | | | | | | | |  | |  |
|  | | | | **Estimate** | **Std. Error** | **T value** | | | | **Pr(>\|t\|)** | | | | | |
| (Intercept) | | | | -0.3196 | 2.3072 | -0.139 | | | | 0.890 | | | | | |
| SamplingPeriod20-21 | | | | 1.1880 | 2.3114 | 0.514 | | | | 0.608 | | | | | |
|  |  | **Approximate Significance of Smooth Terms** | | | | | | | | | | |  | |  |
|  | | | | **edf** | **Ref.df.** | **F** | | | | **p-value** | | | | | |
| s(SIC): SamplingPeriod19-20 | | | | 1.692 | 1.905 | 2.032 | | | | 0.08910 . | | | | | |
| s(SIC): SamplingPeriod20-21 | | | | 1.854 | 1.979 | 5.218 | | | | 0.00857 ^**^ | | | | | |
| s(month): SamplingPeriod19-20 | | | | 1.918 | 1.993 | 76.076 | | | | < 2e-16 ^***^ | | | | | |
| s(month): SamplingPeriod20-21 | | | | 1.995 | 2.000 | 116.413 | | | | < 2e-16 ^***^ | | | | | |
| **Signif.codes: 0 ‘^***^’ 0.001 ‘^**^’ 0.01 ‘^*^’ 0.05 ‘^.^’ 0.1‘’ 1** | | | | | | | |  |  | | |  | |  | |

***Table B11.*** *Model output for effects of Sea Ice Concentration (SIC) and month on song presence at M2 (n=439).*

| **Family** | | | **Link Function** | **Formula** | | | **Adjusted R^2^** | | | | **Deviance Explained** | | | | |
| --- | --- | --- | --- | --- | --- | --- | --- | --- | --- | --- | --- | --- | --- | --- | --- |
| Quasibinomial | | | Logit | Song ~ s(SIC, by = sampling period,  k = 3) + s(month, by = sampling period,  k = 3) + sampling period | | | 0.647 | | | | 60.6% | | | | |
|  |  | **Parametric Coeffficients** | | | | | | | | | | |  | |  |
|  | | | | **Estimate** | **Std. Error** | **T value** | | | | **Pr(>\|t\|)** | | | | | |
| (Intercept) | | | | -5.0260 | 2.8654 | -1.754 | | | | 0.0801 . | | | | | |
| StudyPeriod20-21 | | | | -0.4387 | 2.9907 | -0.147 | | | | 0.8835 | | | | | |
|  |  | **Approximate Significance of Smooth Terms** | | | | | | | | | | |  | |  |
|  | | | | **edf** | **Ref.df.** | **F** | | | | **p-value** | | | | | |
| s(SIC): SamplingPeriod19-20 | | | | 1.757 | 1.941 | 1.771 | | | | 0.209 | | | | | |
| s(SIC): SamplingPeriod20-21 | | | | 1.972 | 1.999 | 24.070 | | | | < 2e-16 ^***^ | | | | | |
| s(month): SamplingPeriod19-20 | | | | 1.000 | 1.000 | 109.808 | | | | < 2e-16 ^***^ | | | | | |
| s(month): SamplingPeriod20-21 | | | | 1.977 | 1.999 | 21.882 | | | | < 2e-16 ^***^ | | | | | |
| **Signif.codes: 0 ‘^***^’ 0.001 ‘^**^’ 0.01 ‘^*^’ 0.05 ‘^.^’ 0.1‘’ 1** | | | | | | | |  |  | | |  | |  | |


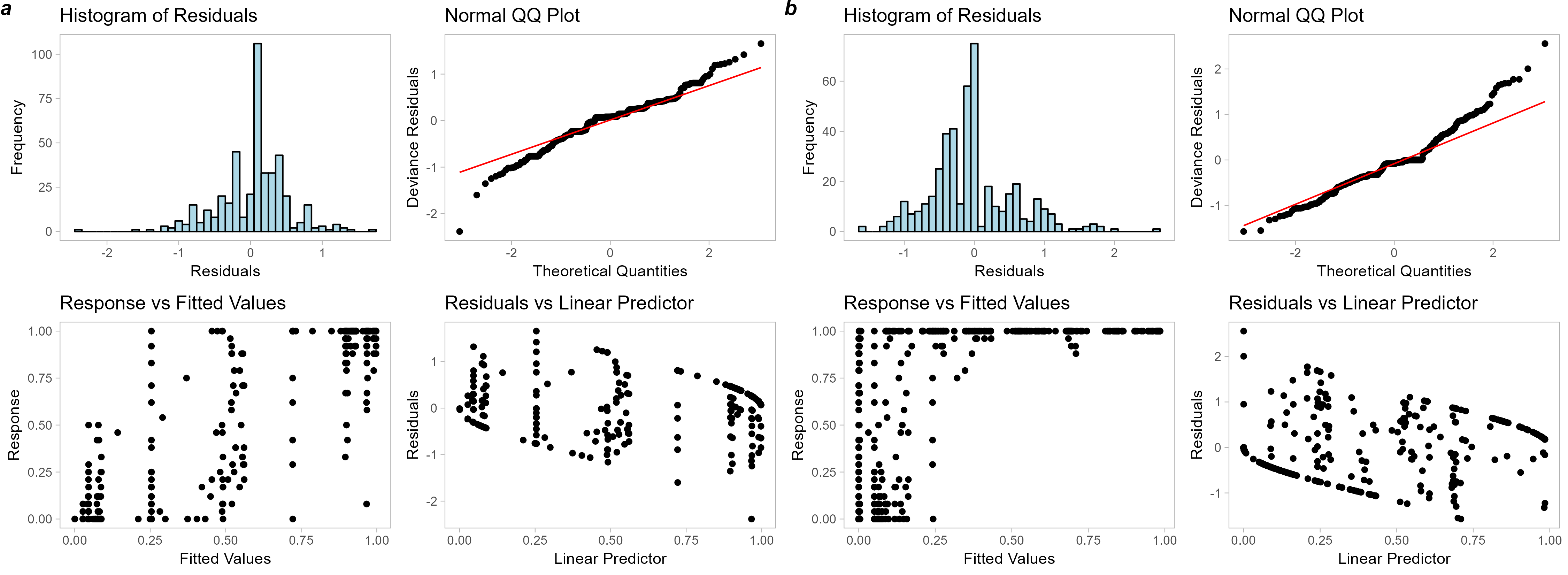


***b***

***a***

***Figure B6.*** *Residual plots for GAMs (Generalized Additive Models) of* ***(a)*** *acoustic presence and* ***(b)*** *song presence of bowhead whales at M2 (n=439). Showing Histogram of Residuals, Q-Q plot, Response vs Fitted Values and Residuals vs Linear Predictor.*
